# Financial time series forecasting using twin support vector regression

**DOI:** 10.1371/journal.pone.0211402

**Published:** 2019-03-13

**Authors:** Deepak Gupta, Mahardhika Pratama, Zhenyuan Ma, Jun Li, Mukesh Prasad

**Affiliations:** 1 Department of Electronics and Computer Engineering, National Institute of Technology, Arunachal Pradesh, India; 2 School of Computer Science and Engineering, Nanyang Technological University, Singapore, Singapore; 3 School of Mathematics and System Sciences, Guangdong Polytechnic Normal University, Guangzhou, China; 4 Centre for Artificial Intelligence, School of Software, Faculty of Engineering and Technology, University of Technology Sydney, Sydney, Australia; Pablo de Olavide University, SPAIN

## Abstract

Financial time series forecasting is a crucial measure for improving and making more robust financial decisions throughout the world. Noisy data and non-stationarity information are the two key factors in financial time series prediction. This paper proposes twin support vector regression for financial time series prediction to deal with noisy data and nonstationary information. Various interesting financial time series datasets across a wide range of industries, such as information technology, the stock market, the banking sector, and the oil and petroleum sector, are used for numerical experiments. Further, to test the accuracy of the prediction of the time series, the root mean squared error and the standard deviation are computed, which clearly indicate the usefulness and applicability of the proposed method. The twin support vector regression is computationally faster than other standard support vector regression on the given 44 datasets.

## Introduction

For the last two decades in the machine learning area, support vector machines (SVMs) have been a computationally powerful kernel-based tool for various classification problems, such as pattern recognition and regression problems and function approximations [1]. It has the advantages over other methods, such as artificial neural networks (ANN), which focus on minimizing the empirical risk in the training phase, whereas SVM was developed on the structural risk minimization principle [1], which minimizes the upper bound on the generalization error. Another advantage of SVM is that it forms a convex optimization problem, a single large quadratic programming problem (QPP) that yields a unique global solution. The SVM has been applied in many fields to solve various well-known real-world problems ranging from image classification [2], remote sensing image classification [3], text characterization [4], biomedicine [5, 6], time series prediction [7, 8] and business prediction [9], which clearly justify its popularity.

To obtain an optimal regressor function for a given set of training data, support vector regression (SVR) was introduced by Vapnik [1], where training data points are in the input space or in a higher dimensional space via kernel mapping. The SVR has the advantage of better generalization performance than the other regression methods. However, standard SVM has a drawback in that it optimizes a computationally expensive cost function for large-scale datasets that have high training costs, i.e., O(*m*^3^), where *m* is the number of training samples. Due to this high training cost, it is not easy to find the optimal parameters from a large set of parameters. To address this issue, different variants of SVM have been proposed, such as chunking and decomposition methods [10, 11], exact SVM training algorithm SMO [12], approximate SVM training algorithms [13–15] and LS-SVM [16, 17].

Mangasarian and Wild [18] suggested a new method for binary classification as a generalized eigenvalue proximal support vector machine (GEPSVM) based on two nonparallel hyperplanes. To find the nonparallel hyperplanes, GEPSVM solves two eigenvalue problems based on the size of the input space dimensions. The GEPSVM outperforms the standard SVM in terms of computational speed and accuracy. Similarly, in the spirit of GEPSVM, twin support vector machine (TWSVM) has recently been proposed [19] for binary classification problems that consist of two nonparallel planes, for example, where each plane is closer to the data points of one of the two classes and as far as possible from the data points of the other class. In TWSVM, two QPPs of smaller size are solved to obtain two nonparallel hyperplanes instead of a QPP of large size. This strategy gives TWSVM good generalization ability, making it better than GEPSVM and approximately four times faster than the standard SVM. The main difference between GEPSVM and TWSVM is that GEPSVM solves two generalized eigenvalue problems to obtain the hyperplanes because TWSVM solves two related SVM-type problems to obtain the hyperplanes. Peng [20] recently proposed a twin support vector regression technique based on TWSVM in which an unknown regressor function is generated by the construction of nonparallel insensitive up and down bound functions. In this case, it solves a pair of two smaller sized QPPs unlike the large QPP solved in the case of SVR. To find the solution to this problem through machine learning approaches, various methods have been applied, such as artificial neural networks [21], statistical learning [22], fuzzy logic [23–26], neural networks [27–29], evolutionary algorithms [30] and hidden Markov models [31]. Eugene et al. [32], estimated that the factors for high expected returns that are due to future price increases are only offset through the decrementing of the current price. Therefore, expected returns based on the variable time generate temporary subsets of different prices. Lewellen et al. [33] proposed an approach for testing the prediction of aggregate financial ratios, named predictive regression, on small-scale sample biases. Goh et al. [34] tried to find the relationship between the U.S. and Chinese economic variables and predicted the economic variable for each country that justifies which country’s economic variables are greater than others. In 2017, Shen et al. [35] presented a novel method for predicting the Chinese stock returns for different asset values using the Baidu index. Similarly, Li et al. (2018) [36] found that idiosyncratic volatility significantly grows when internet stock message boards are already built up.

The prediction of stock market indices has been the focus of interest from the day the stock market came into existence. Researchers have several goals and motivations for trying to predict stock market prices. One of the motivations could be to make life easier and more luxurious. Many investment professionals, along with researchers, are trying to find a superior system that will yield high returns in terms of financial gain. There has been considerable work performed to predict the behavior of the stock market. To perform the financial time series prediction, various parameters are involved: (a) price of the last trade performed during the day, (b) total number of commodities traded during the day, and (c) lowest and highest traded price [37]. Because of these parameters, the nonlinearity and uncertainty involved in the prediction of financial time series forecasting, this paper proposes TSVR to address these situations. To determine the effectiveness of TSVR on financial time series datasets, first, this paper discusses the formulation of TSVR and then the performance of the numerical experiments for various financial datasets. The experimental results of TSVR are compared with the standard SVR formulation with accuracy in terms of average RMSE and training time.

The remainder of this paper is organized as follows: Sections 2 and 3 discuss the formulation of SVR and TSVR, respectively. Section 4 shows the experimental results on different financial time series datasets of TSVR and comparison results with SVR. Finally, conclusions are drawn in section 5.

## Support vector regression

This section describes the standard formulation of support vector regression (SVR). Assume that a set of training samples is {(*x*_1_,*y*_1_)}_*i* = 1,2,…,*m*_ where *x*_*i*_ = (*x*_*i*1_,*x*_*i*2_,…,*x*_*in*_)^*t*^∈*R*^*n*^ is the input example and *y*_*i*_∈*R* is the target value for *i* = 1,2,…,*m*, where *m* corresponds to input training samples. Let matrix *D*∈*R*^*m*×*n*^ denote the input examples where $x_{i}^{t}$ is the *i*-th row and *y* = (*y*_1_,…,*y*_*m*_)^*t*^ is the vector of observed values. The main goal of SVR is to approximate the regression function *f*(.) in the form

$$f(x)=x^{t}w+b$$ (1)

where unknowns *w* is the vector and *b* is a scalar value.

Vapnik [1] suggested the formulations of SVR by introducing the *ε*-insensitive loss function and determining the unknown variables *w* and *b* by solving the following QPP:

$$\underset{(w,b,\xi_{1},\xi_{2})\in R^{n+1+m+m}}{\min}\frac{1}{2}w^{t}w+C(e^{t}\xi_{1}+e^{t}\xi_{2}),$$

subject to:

$$y_{i}-x_{i}^{t}w-b\leq \varepsilon +\xi_{1i},$$

$$x_{i}^{t}w+b-y_{i}\leq \varepsilon +\xi_{2i}$$

and

$$\xi_{1i}\geq 0,\;\xi_{2i}\geq 0\;\mathrm{for}\;i=1,2,\ldots ,m$$ (2)

where *ξ*_1_ = (*ξ*_1*i*_,…,*ξ*_1*m*_)^*t*^, *ξ*_2_ = (*ξ*_21_,…,*ξ*_2*m*_)^*t*^ are slack variables in vector form, and *C*>0 and *ε*>0 denote the input parameters.

Here, the solution of the above problem is obtained by introducing Lagrange multipliers

$$\underset{\lambda_{1},\lambda_{2}\in R^{m}}{\min}\frac{1}{2}\sum_{i,j=1}^{m}{\left(\lambda_{1i}-\lambda_{2i}\right)}^{t}x_{i}^{t}x_{j}(\lambda_{1j}-\lambda_{2j})+\varepsilon \sum_{i=1}^{m}\left(\lambda_{1i}+\lambda_{2i}\right)-\sum_{i=1}^{m}y_{i}(\lambda_{1i}-\lambda_{2i})$$

subject to:

$$\sum_{i=1}^{m}\left(\lambda_{1i}-\lambda_{2i}\right)=0$$

$$0\leq \lambda_{1},\lambda_{2}\leq Ce,$$ (3)

where the Lagrange multipliers are *λ*_1_ = (*λ*_11_,…,*λ*_1*m*_)^*t*^ and *λ*_2_ = (*λ*_21_,…,*λ*_2*m*_)^*t*^ in *R*^*m*^, which give the solution to the above quadratic problem. Here, nonzero values of Lagrangian multipliers, which are known as support vectors in Eq (3) are useful for predicting the regression function, which is defined for any *x*∈*R*^*n*^ as

$$f(x)=\sum_{i=1}^{m}\left(\lambda_{1i}-\lambda_{2i}\right)(x^{t}x_{i})+b$$ (4)

For a nonlinear regressor, the input data maps to a higher dimensional feature space using a kernel function *k* (.,.) which is defined by the Gaussian kernel as *k*(*x*_*i*_,*x*_*j*_) = exp(−*μ*‖*x*_*i*_−*x*_*j*_‖^2^) for *i*, *j* = 1,2,…,*m* and *μ* is a parameter. The nonlinear case can be obtained as

$$\underset{\lambda_{1},\lambda_{2}\in R^{m}}{\min}\frac{1}{2}\sum_{i,j=1}^{m}{\left(\lambda_{1i}-\lambda_{2i}\right)}^{t}k(x_{i},x_{j})(\lambda_{1j}-\lambda_{2j})+\varepsilon \sum_{i=1}^{m}\left(\lambda_{1i}+\lambda_{2i}\right)-\sum_{i=1}^{m}y_{i}(\lambda_{1i}-\lambda_{2i})$$

subject to:

$$\sum_{i=1}^{m}\left(\lambda_{1i}-\lambda_{2i}\right)=0$$

$$0\leq \lambda_{1},\lambda_{2}\leq Ce,$$ (5)

The nonlinear prediction function *f* (.) is given by finding the value of *λ*_1_ and *λ*_2_ from the solution of the problem mentioned in Eq (5) for any *x*∈*R*^*n*^,

$$f(x)=\sum_{i=1}^{m}\left(\lambda_{1i}-\lambda_{2i})k(x,x_{i}\right)+b$$

## Twin support vector machine

To further improve the generalization performance and training time of SVR, a new approach was discussed by Peng [20], termed TSVR. The TSVR constructs a pair of nonparallel hyperplanes such that one of the hyperplanes determines the *ε*-insensitive downbound *f*_1_(*x*) = *x*^*t*^*w*_1_+*b*_1_ and another *ε*-insensitive upbound function *f*_2_(*x*) = *x*^*t*^*w*_2_+*b*_2_ to identify the end regression function. The TSVR solves a pair of smaller QPPs of *m* constraints to identify the solution instead of solving a single large QPP with a 2 m number of constraints.

The formulation of TSVR determines the regression function by the following pair of constrained QPPs as:

$$\min\frac{1}{2}\|y-e\varepsilon_{1}-\left(Dw_{1}+eb_{1}\right){\|}^{2}+C_{1}e^{t}\xi$$

subject to:

$$y-\left(Dw_{1}+eb_{1}\right)\geq e\varepsilon_{1}-\xi ,\;\xi \geq 0$$ (6)

$$\min\frac{1}{2}\|y+e\varepsilon_{2}-\left(Dw_{2}+eb_{2}\right){\|}^{2}+C_{2}e^{t}\eta$$

subject to:

$$\left(Dw_{2}+eb_{2}\right)-y\geq e\varepsilon_{2}-\eta ,\;\eta \geq 0$$ (7)

where *C*_1_,*C*_2_>0 and *ε*_1_,*ε*_2_≥0 denote input parameters, *ξ* = (*ξ*_1_,…*ξ*_*m*_)^*t*^ and *η* = (*η*_1_,…*η*_*m*_)^*t*^ denote the vector of slack variables.

To find the solution of the above primal-based QPPs shown in Eqs (6) and (7), we convert the QPPs into dual forms by using the Lagrange multipliers *λ*_1_ = (*λ*_11_,…*λ*_1*m*_)^*t*^, *ν*_1_ = (*ν*_11_,…*ν*_1*m*_)^*t*^ and *λ*_2_ = (*λ*_21_,…*λ*_2*m*_)^*t*^, *ν*_2_ = (*ν*_21_,…*ν*_2*m*_)^*t*^. The Lagrangian functions of Eqs (6) and (7) are given by Eqs (8) and (9), respectively.

$$L_{1}\left(w_{1},b_{1},\xi ,\lambda_{1},\nu_{1}\right)=\frac{1}{2}\|y-e\varepsilon_{1}-\left(Dw_{1}+eb_{1}\right){\|}^{2}+C_{1}e^{t}\xi -\lambda_{1}\left(y-\left(Dw_{1}+eb_{1}\right)-e\varepsilon_{1}+\xi \right)-\nu_{1}^{t}\xi$$ (8)

$$L_{2}\left(w_{2},b_{2},\eta ,\lambda_{2},\nu_{2}\right)=\frac{1}{2}\|y+e\varepsilon_{2}-\left(Dw_{2}+eb_{2}\right){\|}^{2}+C_{2}e^{t}\eta -\lambda_{2}\left(\left(Dw_{2}+eb_{2}\right)-y-e\varepsilon_{2}+\eta \right)-\nu_{2}^{t}\eta$$ (9)

By applying the KKT conditions for the Lagrangian function as shown in Eq (8), we obtain:

$$-D^{t}\left(y-Dw_{1}-eb_{1}-e\varepsilon_{1}\right)+D^{t}\lambda_{1}=0,$$ (10)

$$-e^{t}\left(y-Dw_{1}-eb_{1}-e\varepsilon_{1}\right)+e^{t}\lambda_{1}=0,$$ (11)

$$C_{1}e-\lambda_{1}-\nu_{1}=0,$$ (12)

$$y-\left(Dw_{1}+eb_{1}\right)\geq e\varepsilon_{1}-\xi ,\;\xi \geq 0,$$ (13)

$$\lambda_{1}^{t}\left(y-\left(Dw_{1}+eb_{1}\right)\geq e\varepsilon_{1}-\xi \right)=0,\;\lambda_{1}\geq 0,$$ (14)

$$\nu_{1}^{t}\xi =0,\;\nu_{1}\geq 0,$$ (15)

Since *ν*_1_≥0, we have

$$0\leq \lambda_{1}\leq C_{1}e.$$ (16)

Similarly, for the Lagrangian function as shown in Eq (9), we obtain

$$-D^{t}\left(y-Dw_{2}-eb_{2}+e\varepsilon_{2}\right)-D^{t}\lambda_{2}=0,$$ (17)

$$-e^{t}\left(y-Dw_{2}-eb_{2}+e\varepsilon_{2}\right)-e^{t}\lambda_{2}=0,$$ (18)

$$C_{2}e-\lambda_{2}-\nu_{2}=0,$$ (19)

$$\left(Dw_{2}+eb_{2}\right)-y\geq e\varepsilon_{2}-\eta ,\;\eta \geq 0,$$ (20)

$$\lambda_{2}^{t}\left(\left(Dw_{2}+eb_{2}\right)-y\geq e\varepsilon_{2}-\eta \right)=0,\;\lambda_{2}\geq 0,$$ (21)

$$\nu_{2}^{t}\eta =0,\;\nu_{2}\geq 0,$$ (22)

Since *ν*_2_≥0, we have

$$0\leq \lambda_{2}\leq C_{2}e.$$ (23)

Combining Eq (10) with Eq (11) and Eq (17) with Eq (18), we obtain

$$-\left[\begin{array}{c} D^{t}\\ e^{t} \end{array}\right]\left\{\left(y-e\varepsilon_{1}\right)-\left[\begin{array}{cc} D & e \end{array}\right]\left[\begin{array}{c} w_{1}\\ b_{1} \end{array}\right]\right\}+\left[\begin{array}{c} D^{t}\\ e^{t} \end{array}\right]\lambda_{1}=0$$ (24)

$$-\left[\begin{array}{c} D^{t}\\ e^{t} \end{array}\right]\left\{\left(y+e\varepsilon_{2}\right)-\left[\begin{array}{cc} D & e \end{array}\right]\left[\begin{array}{c} w_{2}\\ b_{2} \end{array}\right]\right\}-\left[\begin{array}{c} D^{t}\\ e^{t} \end{array}\right]\lambda_{2}=0$$ (25)

Let us define,

$$S=\left[D\;e\right],\;u_{1}={\left[w_{1}^{t}\;b_{1}\right]}^{t},u_{2}={\left[w_{2}^{t}\;b_{2}\right]}^{t},f_{1}=y-e\varepsilon_{1},\;f_{2}=y+e\varepsilon_{2},$$ (26)

and then we have,

$$-S^{t}f_{1}+S^{t}Su_{1}+S^{t}\lambda_{1}=0,$$

i.e.,

$$u_{1}={\left(S^{t}S\right)}^{-1}S^{t}\left(f_{1}-\lambda_{1}\right).$$ (27)

and

$$-S^{t}f_{2}+S^{t}Su_{2}-S^{t}\lambda_{2}=0,$$,

i.e.,

$$u_{2}={\left(S^{t}S\right)}^{-1}S^{t}\left(f_{2}+\lambda_{2}\right).$$ (28)

Here, note that *S*^*t*^*S* is positive semidefinite, but to overcome the situation in which its inverse does not exist, *σI* is introduced as a regularization term, so that (*S*^*t*^*S*+*σI*) becomes positive definite where *σ* is a very small positive number, such as *σ = Ie-7*. Thus, we have

$$u_{1}={\left(S^{t}S+\sigma I\right)}^{-1}S^{t}\left(f_{1}-\lambda_{1}\right)$$ (29)

$$u_{2}={\left(S^{t}S+\sigma I\right)}^{-1}S^{t}\left(f_{2}+\lambda_{2}\right)$$ (30)

Substituting Eq (29) into the primal Lagrangian function Eq (8) and using Eqs (13) to (16), the dual problem of Eq (6) is obtained as

$$\max-\frac{1}{2}\lambda_{1}^{t}S{\left(S^{t}S\right)}^{-1}S^{t}\lambda_{1}+f_{1}^{t}S{\left(S^{t}S\right)}^{-1}S^{t}\lambda_{1}-f_{1}^{t}\lambda_{1}$$

subject to:

$$0\leq \lambda_{1}\leq eC_{1}$$ (31)

Similarly, substituting Eq (30) into the primal Lagrangian function Eq (9) and using Eq (20) to (23), the dual problem of Eq (7) is obtained as

$$\max-\frac{1}{2}\lambda_{2}^{t}S{\left(S^{t}S\right)}^{-1}S^{t}\lambda_{2}-f_{2}^{t}S{\left(S^{t}S\right)}^{-1}S^{t}\lambda_{2}+f_{2}^{t}\lambda_{2}$$

subject to:

$$0\leq \lambda_{2}\leq eC_{2}$$ (32)

The vectors *λ*_1_ and *λ*_2_ are calculated by solving the dual QPPs Eqs (31) and (32). Finally, in the output for any data point *x*∈*R*^*n*^, the end regressor *f*(.) is given by:

$$f\left(x\right)=\frac{1}{2}\left(f_{1}\left(x\right)+f_{2}\left(x\right)\right).$$ (33)

To extend TSVR to a nonlinear case, TSVR finds the regression function by solving the following primal problems:

$$\min\frac{1}{2}\|y-e\varepsilon_{1}-\left(K(D,D^{t})w_{1}+eb_{1}\right){\|}^{2}+C_{1}e^{t}\xi$$

subject to:

$$y-\left(K(D,D^{t})w_{1}+eb_{1}\right)\geq e\varepsilon_{1}-\xi ,\;\xi \geq 0$$ (34)

and

$$\min\frac{1}{2}\|y+e\varepsilon_{2}-\left(K(D,D^{t})w_{2}+eb_{2}\right){\|}^{2}+C_{2}e^{t}\eta$$

subject to:

$$\left(K(D,D^{t})w_{2}+eb_{2}\right)-y\geq e\varepsilon_{2}-\eta ,\;\eta \geq 0$$ (35)

where the kernel matrix *K*(*D*,*D*^*t*^) of order *m* whose (*i*, *j*) element is given by *K*(*D*,*D*^*t*^)_*ij*_ = *k*(*x*_*i*_,*x*_*j*_)∈*R*, and where *k*(*x*_*i*_,*x*_j_) is a nonlinear kernel function. For a vector *x*∈*R*^*n*^, we define

$$k(x^{t},D^{t})=(k(x,x_{1}),\ldots ,k(x,x_{m}))$$

in a similar manner, the dual formulations of QPPs Eqs (34) and (35) are given by Eqs (36) and (37), respectively.

$$\max-\frac{1}{2}\lambda_{1}^{t}T{\left(T^{t}T\right)}^{-1}T^{t}\lambda_{1}+f_{1}^{t}T{\left(T^{t}T\right)}^{-1}T^{t}\lambda_{1}-f_{1}^{t}\lambda_{1}$$

subject to:

$$0\leq \lambda_{1}\leq eC_{1}$$ (36)

and

$$\max-\frac{1}{2}\lambda_{2}^{t}T{\left(T^{t}T\right)}^{-1}T^{t}\lambda_{2}-f_{2}^{t}T{\left(T^{t}T\right)}^{-1}T^{t}\lambda_{2}+f_{2}^{t}\lambda_{2}$$

subject to:

$$0\leq \lambda_{2}\leq eC_{2}$$ (37)

where *T* = [*K*(*D*,*D*^*t*^) *e*]. After resolving Eqs (36) and (37), we find the value of *u*_1_ and *u*_2_ as

$$u_{1}={\left(T^{t}T+\sigma I\right)}^{-1}T^{t}\left(f_{1}-\lambda_{1}\right)$$ (38)

$$u_{2}={\left(T^{t}T+\sigma I\right)}^{-1}T^{t}\left(f_{2}+\lambda_{2}\right)$$ (39)

Finally, for any data sample *x*∈*R*^*n*^, the end regression function *f*(.) is given by:

$$f\left(x\right)=\frac{1}{2}\left(\left[K(x^{t},D^{t})\;1](u_{1}+u_{2}\right)\right)$$ (40)

## Numerical experiments

In this section, various numerical experiments are conducted to test the generalization performance and the computational efficiency of the TSVR on standard datasets and compared with SVR. This paper considered 44 benchmark datasets and divided them into two groups. The first group has a combination of 24 individual company stocks, and the second group has 20 stock market index datasets from the Yahoo financial website, i.e., http://finance.yahoo.com [38]. Individual company stock datasets are AT&T Inc. (T), Infosys Limited (INFY), Apple, Inc. (AAPL), Facebook, Inc. (FB), Cisco Systems, Inc. (CSCO), Alphabet, Inc. (Goog), Citigroup, Inc. (C), HSBC Holding Plc (HSBC), ICICI Bank, Ltd. (IBN), Royal Bank of Canada (RY), Royal Bank of Scotland (RBS), State Bank of India (SBIN.NS), Punjab National Bank (PNB.NS), International Business Machines Corporation (IBM), Microsoft Corporation (MSFT), Tata Consultancy Services Limited (TCS.BO), Oracle Corporation (ORCL), Bharat Petroleum Corporation Limited (BPCL.NS), Oil India Limited (OIL.NS), Oil and Natural Gas Corporation (ONGC.NS), Royal Dutch Shell Plc (RDS-B), Exxon Mobil Corporation (XOM), Sinopec Shanghai Petrochemical Company Limited (SHI), Hindustan Petroleum Corporation Limited (HINDPETRO.NS) and the stock market index datasets are S&P BSE SENSEX (BSESN), NIFTY 50 (NSEI), CAC 40 (FCHI), ESTX 50 PR.EUR (STOXX50E), KOSPI Composite (KS11), IBEX 35 (IBEX), Nikkei 225 (N225), AEX (AEX), DAX PERFORMANCE (GDAXI), IBOVESPA (BVSP), S&P/TSX Composite (GSPTSE), IPC MEXICO (MXX), SMI PR (SSMI), Dow Jones Industrial Average (DJI), HANG SENG INDEX (HSI), TSEC weighted index (TWII), NASDAQ Composite (IXIC), BEL 20 (BFX), Austrian Traded Index in EUR (ATX), Jakarta Composite Index (JKSE). The details of these datasets are listed in Table 1 and Table 2, respectively.

**Table 1:** Individual stock financial details with their stock exchanges, types and listing abbreviations.

Company name	Registered stock exchange	Listing abbreviation
AT&T Inc.	Equity-NYSE	T
Infosys Limited	Equity-NYSE	INFY
Apple Inc.	Equity-NASDAQ	AAPL
Facebook Inc.	Equity-NASDAQ	FB
Cisco Systems, Inc.	Equity-NASDAQ	CSCO
Alphabet Inc.	Equity-NASDAQ	Goog
Citigroup Inc.	Equity-NYSE	C
HSBC Holding Plc	Equity-NYSE	HSBC
ICICI Bank Ltd.	Equity-NYSE	IBN
Royal Bank of Canada	Equity-NYSE	RY
Royal Bank of Scotland	Equity-NYSE	RBS
State Bank of India	Equity-NSE	SBIN.NS
Punjab National Bank	Equity-NSE	PNB.NS
International Business Machines Corporation	Equity-NYSE	IBM
Microsoft Corporation	Equity-NASDAQ	MSFT
Tata Consultancy Services Limited	Equity-BSE	TCS.BO
Oracle Corporation	Equity-NYSE	ORCL
Bharat Petroleum Corporation Limited	Equity-NSE	BPCL.NS
Oil India Limited	Equity-NSE	OIL.NS
Oil and Natural Gas Corporation	Equity-NSE	ONGC.NS
Royal Dutch Shell Plc	Equity-NYSE	RDS-B
Exxon Mobil Corporation	Equity-NYSE	XOM
Sinopec Shanghai Petrochemical Company Limited	Equity-NYSE	SHI
Hindustan Petroleum Corporation Limited	Equity-NSE	HINDPETRO.NS

**Table 2:** Financial stock market index details with their stock exchanges, types and listing abbreviations.

Stock market index name	Registered stock exchange	Listing abbreviation
S&P BSE SENSEX	Index-Bombay Stock Exchange	BSESN
NIFTY 50	Index-National Stock Exchange	NSEI
CAC 40	Index-Paris Stock Exchange	FCHI
ESTX 50 PR.EUR	Index-Zurich Stock Exchange	STOXX50E
KOSPI Composite Index	Index-Korea Stock Exchange	KS11
IBEX 35.	Index-Madrid Stock Exchange	IBEX
Nikkei 225	Index-Osaka Stock Exchange	N225
AEX-INDEX	Index-Amsterdam Stock Exchange	AEX
DAX PERFORMANCE-INDEX	Index-Xetra, Frankfurt Stock Exchange	GDAXI
IBOVESPA	Index-Sao Paolo Stock Exchange	BVSP
S&P/TSX Composite index	Index-Toronto Stock Exchange	GSPTSE
IPC MEXICO	Index-Mexico Stock Exchange	MXX
SMI PR	Index-VTX,SIX Swiss Exchange	SSMI
Dow Jones Industrial Average	Index-New York Stock Exchange	DJI
HANG SENG INDEX	Index-Hong Kong Stock Exchange	HSI
TSEC weighted index	Index-Taiwan Stock Exchange	TWII
NASDAQ Composite	Index-Nasdaq GIDS, American stock exchange	IXIC
BEL 20	Index-Brussels Stock Exchange	BFX
Austrian Traded Index in EUR	Index-Vienna Stock Exchange	ATX
Jakarta Composite Index	Index-Jakarta Stock Exchange	JKSE

All computations are carried out on a PC with Windows 7 OS, with a 32 bit, 3.10 GHz Intel core i5-2400 processor with 4 GB of RAM under the MATLAB R2012b environment. This paper used the MOSEK optimization toolbox to solve the quadratic programming problem in SVR and TSVR formulations, which is taken from http://www.mosek.com [39].

All the datasets are normalized in the following manner so that each feature value lies in [0, 1]:

$${\bar{d}}_{ij}=\frac{d_{ij}-d_{j}^{\min}}{d_{j}^{\max}-d_{j}^{\min}}$$

where ${\bar{d}}_{ij}$ is the normalized value corresponding to *d*_*ij*_ and $d_{j}^{\max}=\max_{i=1}^{m}\left(d_{ij}\right)$ and $d_{j}^{\min}=\min_{i=1}^{m}\left(d_{ij}\right)$ denote the maximum and minimum values of the *j*-th feature of *A*, respectively. To measure the prediction performance, this paper considered the root mean square error (RMSE), which is given by

$$RMSE=\sqrt{\frac{1}{P}\sum_{i=1}^{P}{\left(y_{i}-{\tilde{y}}_{i}\right)}^{2}},$$

where the total number of test samples is denoted by *P*, and ${\tilde{y}}_{i}$ is the predicted value corresponding to the observed values. To construct a nonlinear regressor, we use a Gaussian kernel

$$k(x,y)=\exp(-\mu {\left\|x-y\right\|}^{2})$$

where vector *x*,*y*∈*R*^*m*^ and *μ*>0. The optimal parameter values of *C* = *C*_1_ = *C*_2_ are selected from the sets {10^−5^,…,10^5^} and μ from the set {2^−5^,…,2^5^} for the training using 10-fold cross validation. By using the optimal values, the whole dataset is divided into 10 equal parts at random, out of which one part is used for testing and the remaining parts for the training to obtain the computational test accuracy. Finally, to measure the prediction, the average RMSE of the test accuracies is considered.

### Individual stocks datasets of company

Individual company stocks such as SBIN.NS, PNB.NS, BPCL.NS, OIL.NS, TCS.BO, HINDPETRO.NS, ONGC.NS consist of 735 closing prices, while T, INFY, AAPL, FB, CSCO, Goog, C, HSBC, IBN, RY, RBS, IBM, MSFT, ORCL, RDS-B, XOM, SHI have a total of 751 closing prices starting from 01-01-2015 to 31-12-2017. The current value is predicted by the previous five closing prices.

#### Linear case

**Fig 1:**
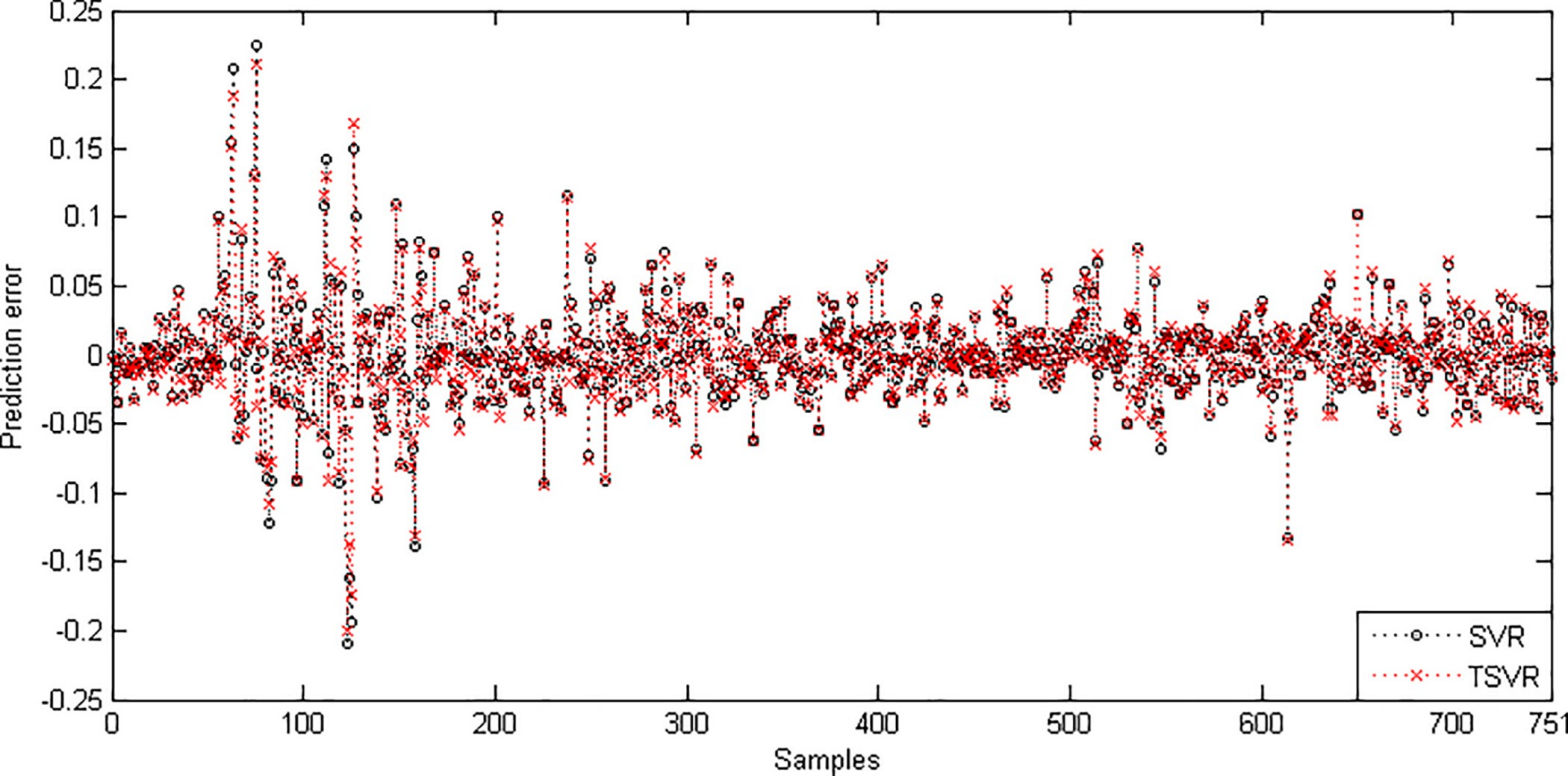
Prediction error plots using a linear kernel on the SHI dataset.

**Fig 2:**
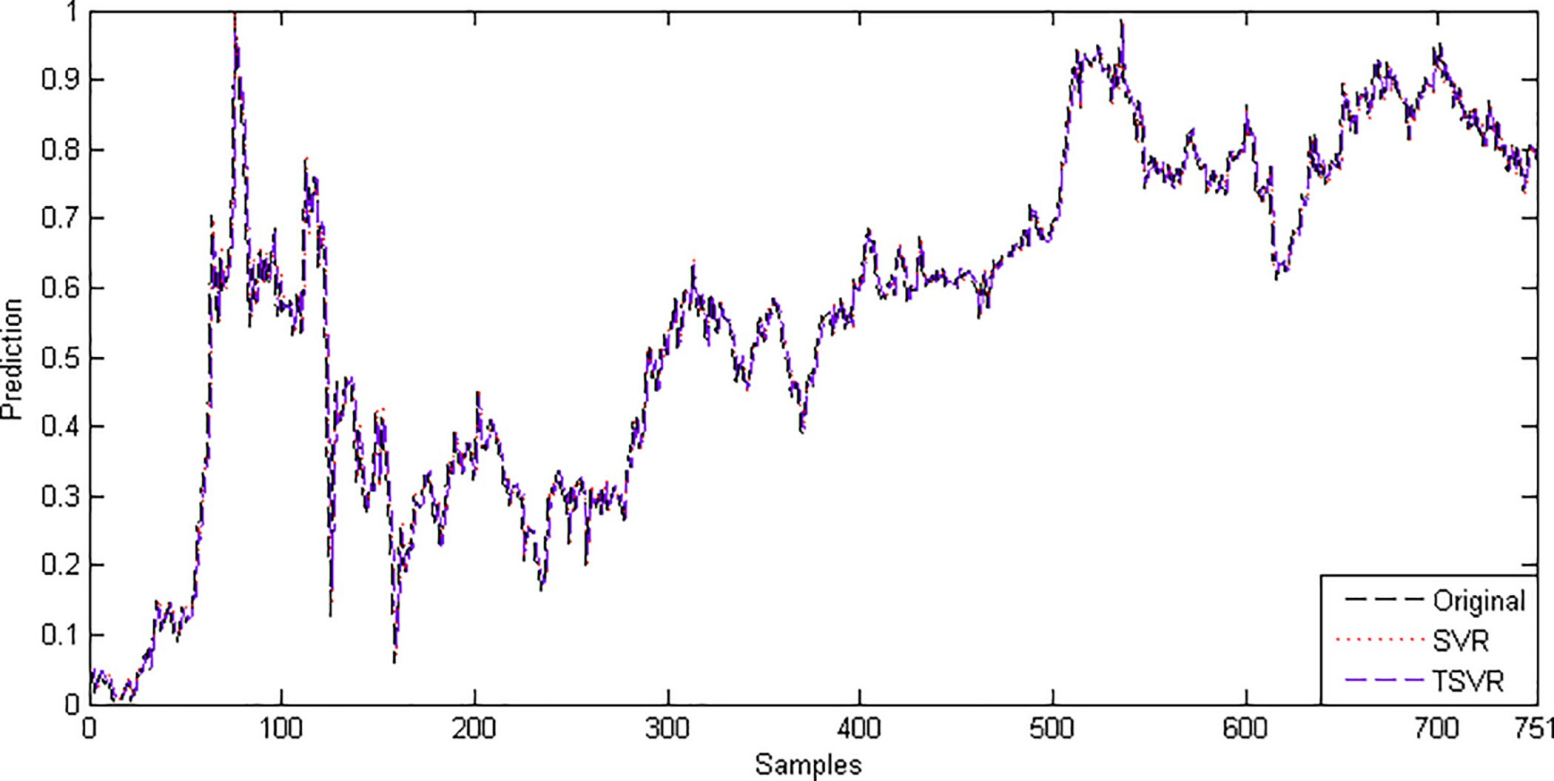
Predicted and actual values using a linear kernel on the SHI dataset.

In the linear case, Table 3 shows the average RMSE for the optimal parameter values with standard deviation and the training time in seconds. Fig 1 shows the absolute prediction error of SVR and TSVR for the linear kernel on the SHI dataset. Fig 2 shows the actual and predicted values of SVR and TSVR for the linear kernel on the SHI dataset. To verify the performance of both algorithms statistically on 24 individual stock datasets, we perform a simple, nonparametric safe test, i.e., the Friedman test with the corresponding post hoc test [40]. For this, the average rank of 24 datasets for the linear case is tabulated in Table 4. The Friedman statistic [40] can be computed under the null hypothesis, as shown in Table 4.

**Table 3:** Performance comparison of TSVR with SVR on individual companies’ stock datasets using a linear kernel. RMSE is used for comparison. Time is used for the training in seconds.

Dataset	SVR (C) Time	TSVR (C1 = C2) Time
**C** **(751X6)**	0.01865+0.00425 (10^-2) 10.9164	0.01866+0.00415 (10^2) 1.37938
**HSBC** **(751X6)**	0.02305+0.00393 (10^-2) 11.2698	0.02306+0.004 (10^1) 1.35164
**IBN** **(751X6)**	0.02234+0.00383 (10^-1) 11.155	0.02232+0.00389 (10^1) 1.30003
**PNB_NS** **(735X6)**	0.02609+0.01228 (10^-1) 10.5834	0.026+0.0123 (10^2) 1.6011
**RY** **(751X6)**	0.0186+0.00467 (10^-1) 11.6193	0.05662+0.10278 (10^5) 1.48767
**RBS** **(751X6)**	0.01923+0.00868 (10^-2) 11.5917	0.01923+0.00869 (10^1) 1.05937
**SBIN_NS** **(735X6)**	0.02872+0.00915 (10^0) 10.885	0.02885+0.00922 (10^1) 1.72065
**AAPL** **(751X6)**	0.01989+0.0037 (10^-3) 11.6285	0.0199+0.00368 (10^2) 1.05923
**AT&T** **(751X6)**	0.03191+0.00567 (10^-2) 11.6016	0.11026+0.25187 (10^5) 1.31281
**CSCO** **(751X6)**	0.02219+0.00506 (10^-1) 11.0575	0.02221+0.00506 (10^2) 0.95512
**FB** **(751X6)**	0.01507+0.00474 (10^-1) 11.1297	0.01506+0.00476 (10^2) 1.23056
**GOOG** **(751X6)**	0.01647+0.00412 (10^-1) 11.1765	0.09118+0.23693 (10^5) 1.24606
**IBM** **(751X6)**	0.02789+0.0057 (10^-2) 10.8029	0.02781+0.00573 (10^2) 0.98122
**INFY** **(751X6)**	0.04022+0.01021 (10^-2) 10.7731	0.04016+0.01022 (10^0) 1.00669
**MSFT** **(751X6)**	0.01601+0.00416 (10^-1) 10.9651	0.01604+0.00411 (10^2) 1.04679
**ORCL** **(751X6)**	0.02717+0.00563 (10^-1) 10.9254	0.02719+0.00563 (10^2) 1.56594
**TCS_BO** **(735X6)**	0.02581+0.0391 (10^-1) 10.387	0.02301+0.03058 (10^-1) 1.01678
**BPCL_NS** **(735X6)**	0.02017+0.00389 (10^-1) 10.3464	0.02012+0.00392 (10^2) 1.23517
**HINDPETRO_NS** **(735X6)**	0.01594+0.00654 (10^-2) 10.8712	0.02444+0.02563 (10^5) 1.12206
**OIL_NS** **(735X6)**	0.02393+0.00654 (10^-2) 10.3806	0.02389+0.00651 (10^1) 1.06545
**ONGC_NS** **(735X6)**	0.02515+0.00599 (10^-1) 10.3224	0.02521+0.00602 (10^2) 1.23187
**RDS_B** **(751X6)**	0.02553+0.00797 (10^-1) 10.8088	0.0255+0.00802 (10^1) 1.34066
**SHI** **(751X6)**	0.03325+0.01405 (10^-2) 10.7923	0.03331+0.01461 (10^2) 1.1263
**XOM** **(751X6)**	0.03385+0.01181 (10^-3) 10.7708	0.0339+0.0119 (10^2) 1.2065

**Table 4:** Average ranks of TSVR with SVR on individual companies’ stocks using a linear and Gaussian kernel.

Dataset	Linear		Non-Linear	
	SVR	TSVR	SVR	TSVR
**C**	1	2	2	1
**HSBC**	1	2	1	2
**IBN**	2	1	2	1
**PNB_NS**	2	1	2	1
**RY**	1	2	2	1
**RBS**	2	1	2	1
**SBIN_NS**	1	2	1	2
**AAPL**	1	2	2	1
**AT&T**	1	2	1	2
**CSCO**	1	2	2	1
**FB**	2	1	2	1
**GOOG**	1	2	2	1
**IBM**	2	1	1	2
**INFY**	2	1	2	1
**MSFT**	1	2	2	1
**ORCL**	1	2	2	1
**TCS_BO**	2	1	2	1
**BPCL_NS**	2	1	2	1
**HINDPETRO_NS**	1	2	2	1
**OIL_NS**	2	1	2	1
**ONGC_NS**	1	2	2	1
**RDS_B**	2	1	2	1
**SHI**	1	2	2	1
**XOM**	1	2	1	2
**Average rank**	1.416667	1.583333	1.791667	1.208333

$$\chi_{F}^{2}=\frac{12\times 24}{2\times (2+1)}\left[(1.416667^{2}+1.583333^{2})-\frac{2\times {\left(2+1\right)}^{2}}{4}\right]≅0.6667$$

$$F_{F}=\frac{(24-1)\times 0.6667}{24\times (2-1)-0.6667}≅0.6572$$

where *F*_*F*_ is distributed according to the *F*-distribution with (1, 23), which has the critical value 4.2793 for the level of significance *α* = 0.05. Here, *F*_*F*_ is lower than the critical value, i.e., 0.6572<4.2793, so there is no significant difference between these two algorithms for the linear case.

#### Nonlinear case

In the nonlinear case, Table 5 shows the average RMSE for the optimal parameter values with the standard deviation and the training time in seconds. From Table 5, we can conclude that TSVR gives better results in 19 cases out of 24 datasets in terms of average RMSE of test accuracy, which signifies the performance of TSVR in comparison to SVR in terms of prediction. Additionally, it shows the superiority of TSVR with respect to SVR in terms of computational time.

**Table 5:** Performance comparison of TSVR with SVR on individual companies’ stock datasets using a Gaussian kernel. RMSE is used for comparison. Time is used for the training in seconds.

Dataset	SVR (C,μ) Time	TSVR (C1 = C2,μ) Time
**C** **(751X6)**	0.0197+0.00459 (10^0,2^-1) 12.8056	0.01925+0.00438 (10^3,2^-2) 1.84222
**HSBC** **(751X6)**	0.02342+0.00369 (10^-1,2^-2) 12.7637	0.02355+0.00378 (10^2,2^-3) 1.46775
**IBN** **(751X6)**	0.02394+0.00612 (10^0,2^-4) 12.6499	0.02252+0.00407 (10^2,2^-5) 1.69161
**PNB_NS** **(735X6)**	0.02619+0.01202 (10^-1,2^-2) 12.1768	0.02598+0.01208 (10^3,2^-5) 1.45579
**RY** **(751X6)**	0.02098+0.00585 (10^0,2^-5) 12.7447	0.01911+0.00521 (10^1,2^-5) 1.70301
**RBS** **(751X6)**	0.01948+0.00876 (10^-1,2^-2) 12.5943	0.01939+0.00868 (10^3,2^-5) 1.5127
**SBIN_NS** **(735X6)**	0.02908+0.00925 (10^-1,2^-2) 11.704	0.02912+0.00981 (10^2,2^-5) 1.46473
**AAPL** **(751X6)**	0.0207+0.00421 (10^-1,2^-2) 12.3965	0.01995+0.00371 (10^2,2^-5) 1.53151
**AT&T** **(751X6)**	0.03185+0.0057 (10^-1,2^-2) 12.3917	0.03192+0.00601 (10^2,2^-5) 1.49755
**CSCO** **(751X6)**	0.02362+0.00534 (10^0,2^-5) 12.3661	0.02243+0.00511 (10^3,2^-5) 1.81103
**FB** **(751X6)**	0.01743+0.00519 (10^0,2^-5) 12.2778	0.01515+0.00465 (10^2,2^-4) 1.79247
**GOOG** **(751X6)**	0.01828+0.00648 (10^-1,2^-2) 12.3224	0.01659+0.00417 (10^2,2^-4) 1.50103
**IBM** **(751X6)**	0.02855+0.00581 (10^0,2^-5) 12.1636	0.21208+0.12217 (10^-3,2^-3) 1.73274
**INFY** **(751X6)**	0.0402+0.01014 (10^0,2^-4) 12.2526	0.04002+0.01014 (10^1,2^-5) 1.69419
**MSFT** **(751X6)**	0.01793+0.00522 (10^0,2^-5) 12.3601	0.01629+0.00434 (10^3,2^-5) 1.74959
**ORCL** **(751X6)**	0.02844+0.00647 (10^-1,2^-5) 12.2863	0.02717+0.00566 (10^2,2^-5) 1.5399
**TCS_BO** **(735X6)**	0.0199+0.02908 (10^-1,2^2) 11.7124	0.01963+0.02914 (10^0,2^1) 1.57151
**BPCL_NS** **(735X6)**	0.0204+0.00377 (10^0,2^-2) 11.7141	0.02023+0.00395 (10^2,2^-5) 1.61242
**HINDPETRO_NS** **(735X6)**	0.01869+0.00916 (10^1,2^-4) 11.8947	0.01607+0.00664 (10^3,2^-3) 1.52778
**OIL_NS** **(735X6)**	0.02512+0.00797 (10^0,2^-2) 11.7162	0.02407+0.0067 (10^2,2^-5) 1.63295
**ONGC_NS** **(735X6)**	0.02644+0.00678 (10^-1,2^-4) 11.7554	0.02581+0.00658 (10^2,2^-5) 1.37471
**RDS_B** **(751X6)**	0.02737+0.01047 (10^-1,2^-4) 12.3922	0.02587+0.00841 (10^1,2^-5) 1.48654
**SHI** **(751X6)**	0.03433+0.01577 (10^-1,2^-4) 12.3041	0.03366+0.01511 (10^2,2^-5) 1.45092
**XOM** **(751X6)**	0.03391+0.01177 (10^0,2^-4) 12.3424	0.03395+0.01186 (10^2,2^-5) 1.7403

Similar to linear case, for individual stocks, the Friedman statistic can be computed under the null hypothesis from Table 4, which shows that both algorithms have a similar performance:

$$\chi_{F}^{2}=\frac{12\times 24}{2\times (2+1)}\left[(1.791667^{2}+1.208333^{2})-\frac{2\times {\left(2+1\right)}^{2}}{4}\right]≅8.1667$$

$$F_{F}=\frac{(24-1)\times 8.1667}{24\times (2-1)-8.1667}≅11.8632$$

where *F*_*F*_ is the distribution according to the *F*-distribution and (1,1×23) = (1, 23) is the degree of freedom. Here, 4.2793 is the critical value of *F*(1,23) for the level of significance at *α* = 0.05. Since the value of *F*_*F*_ = 11.8632>4.2793, we reject the null hypothesis. Furthermore, we performed pairwise comparisons using the Nemenyi post hoc test of all reported methods and verified the significant difference between their average ranks by computing the critical difference (CD) at *p* = 0.10. The difference between their ranks should be at least $1.645\sqrt{\frac{2\times (2+1)}{6\times 24}}\approx 0.3358$.

**Fig 3:**
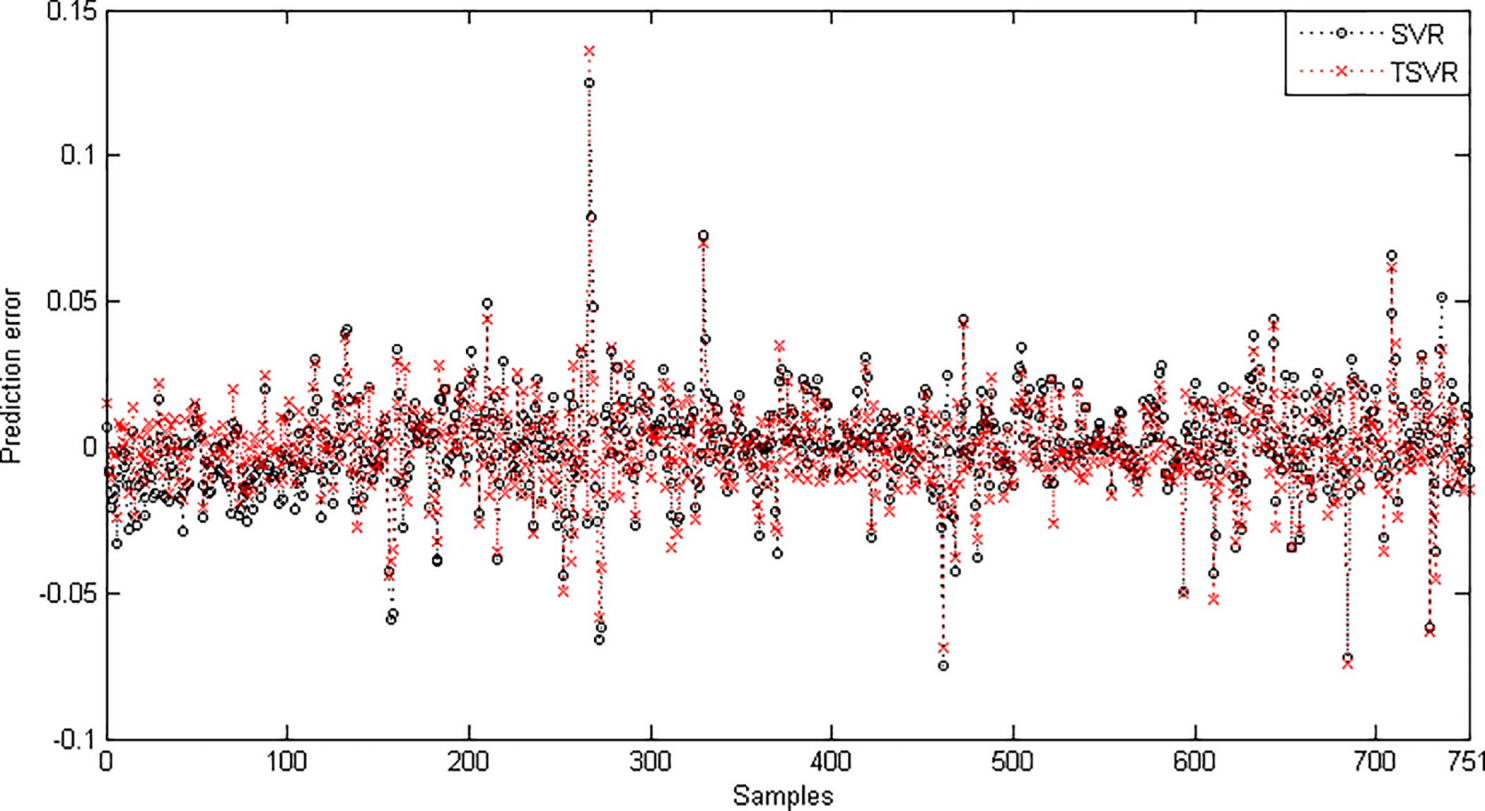
Prediction error plots using a Gaussian kernel on the FB dataset.

**Fig 4:**
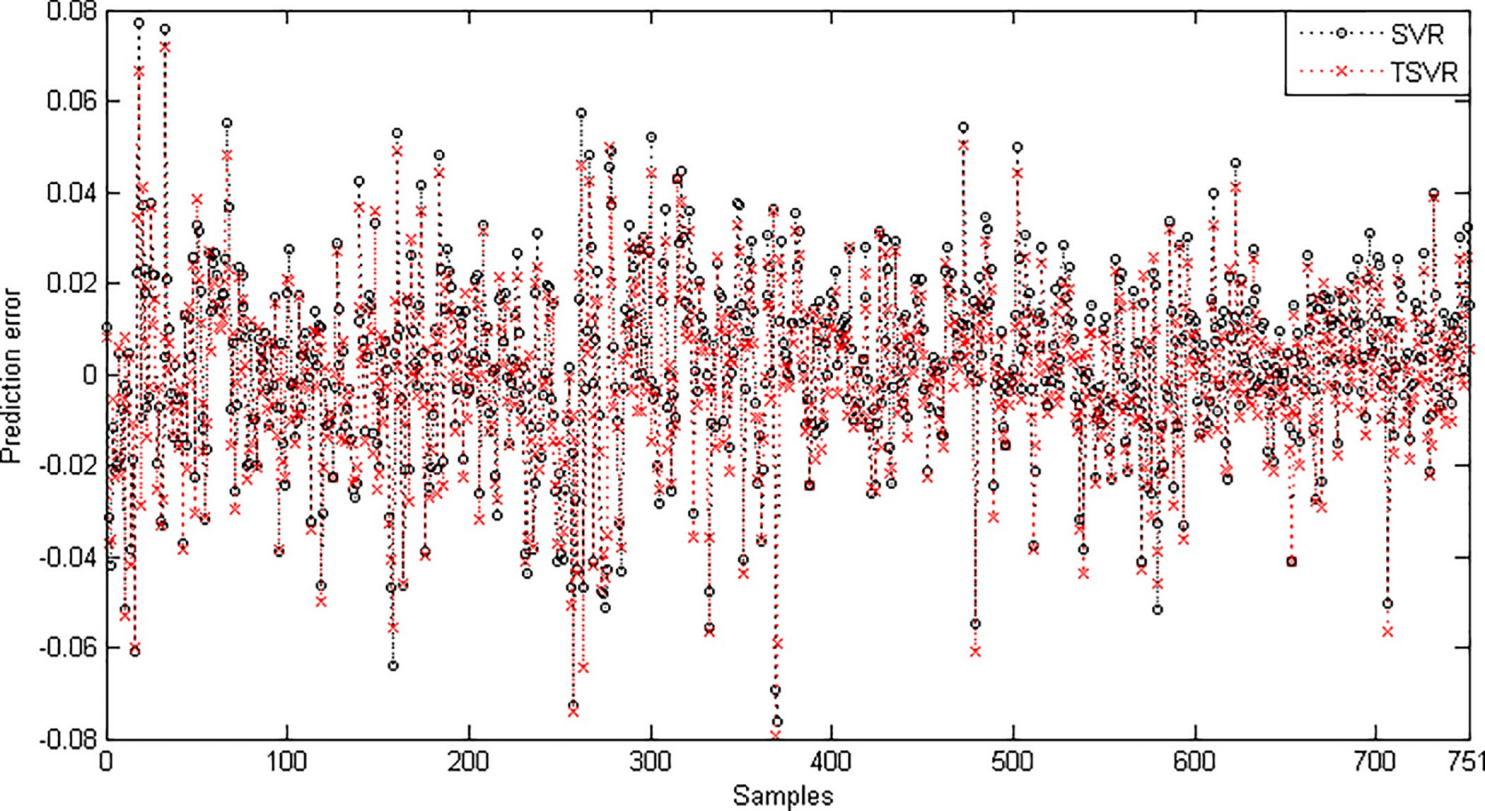
Prediction error plots using a Gaussian kernel on the RY dataset.

**Fig 5:**
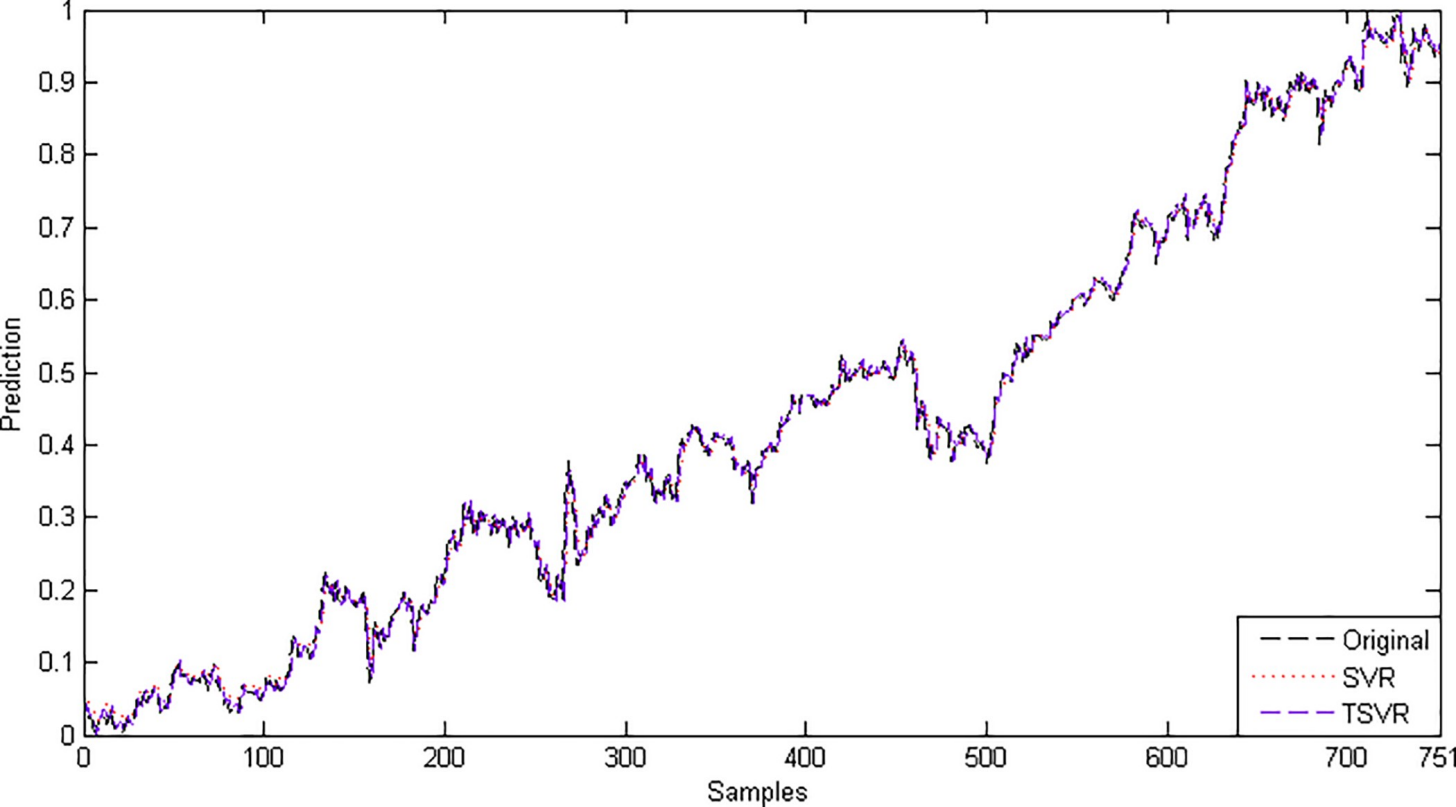
Predicted and actual values using a Gaussian kernel on the FB dataset.

**Fig 6:**
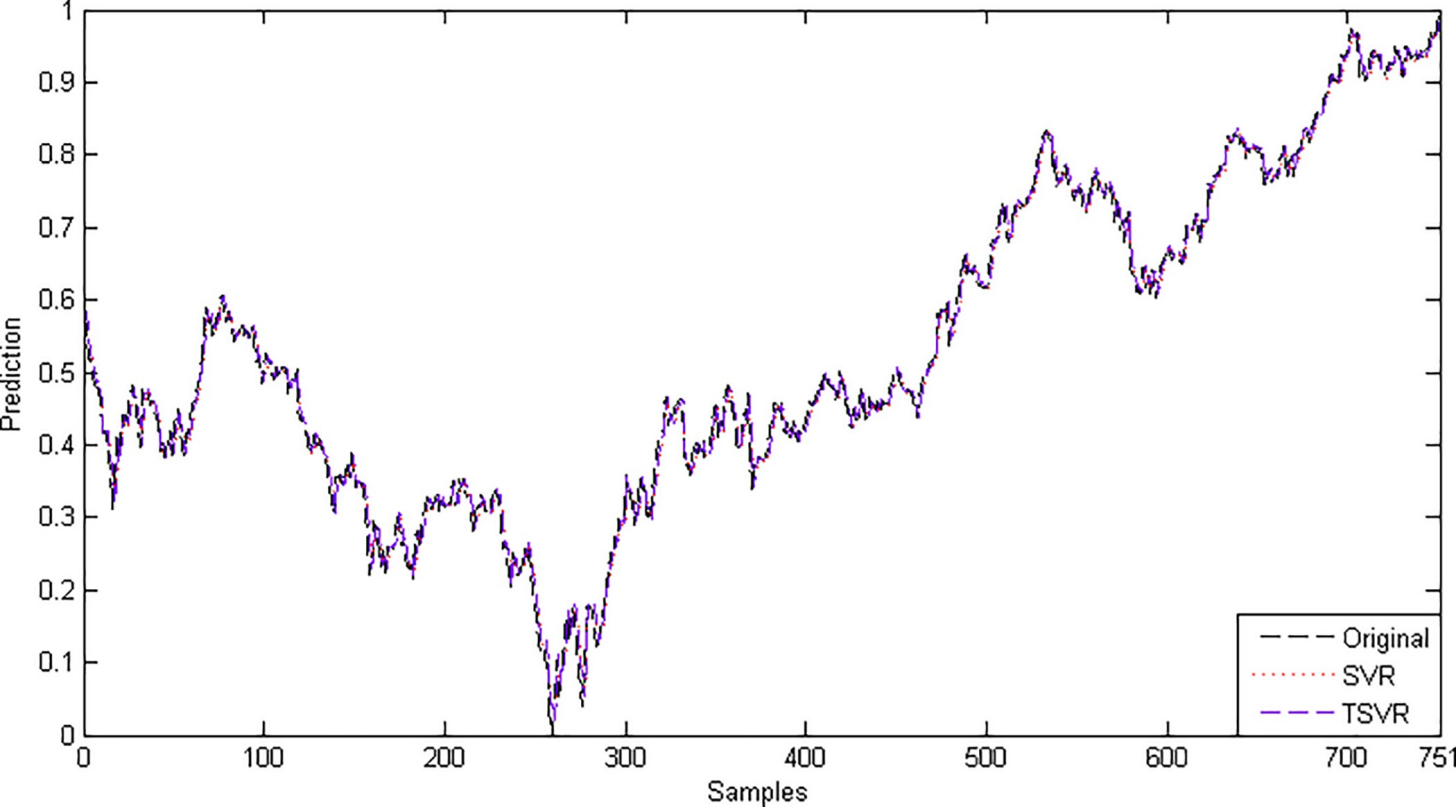
Predicted and actual values using a Gaussian kernel on the RY dataset.

Since the difference between the average ranks of TSVR with SVR (1.791667−1.208333 = 0.583334) is greater than 0.3358, we conclude that TSVR is significantly better than SVR for individual stock datasets. For the non-linear case, the absolute prediction error of SVR and TSVR is shown in Figs 3 and 4 for the FB and RY datasets, respectively. Additionally, the actual and predicted values of SVR and TSVR are plotted in Figs 5 and 6 for the FB and RY datasets, respectively. It can easily be observed that TSVR is in close agreement with the observed values compared to SVR.

### Stock market index datasets

Stock market index datasets such as BSESN and HSI consist of 733 closing prices, while DJI and IXIC have 751 closing prices; the FCHI and IBEX datasets consist of 763 closing prices; the JKSE and TWII datasets consist of 724 closing prices; MXX and SSMI have 750 closing points; AEX consists of 763 closing points; ATX consists of 737 closing points; BFX consists of 762 closing points; BVSP consists of 738 closing points and GDAXI, GSPTSE, KS11, N225, NSEI, STOXX50E consist of 755, 748,728, 732, 731, 745 closing points, respectively, from 01-01-2015 to 31-12-2017. The current value is predicted by using the previous five closing prices.

#### Linear case

**Fig 7:**
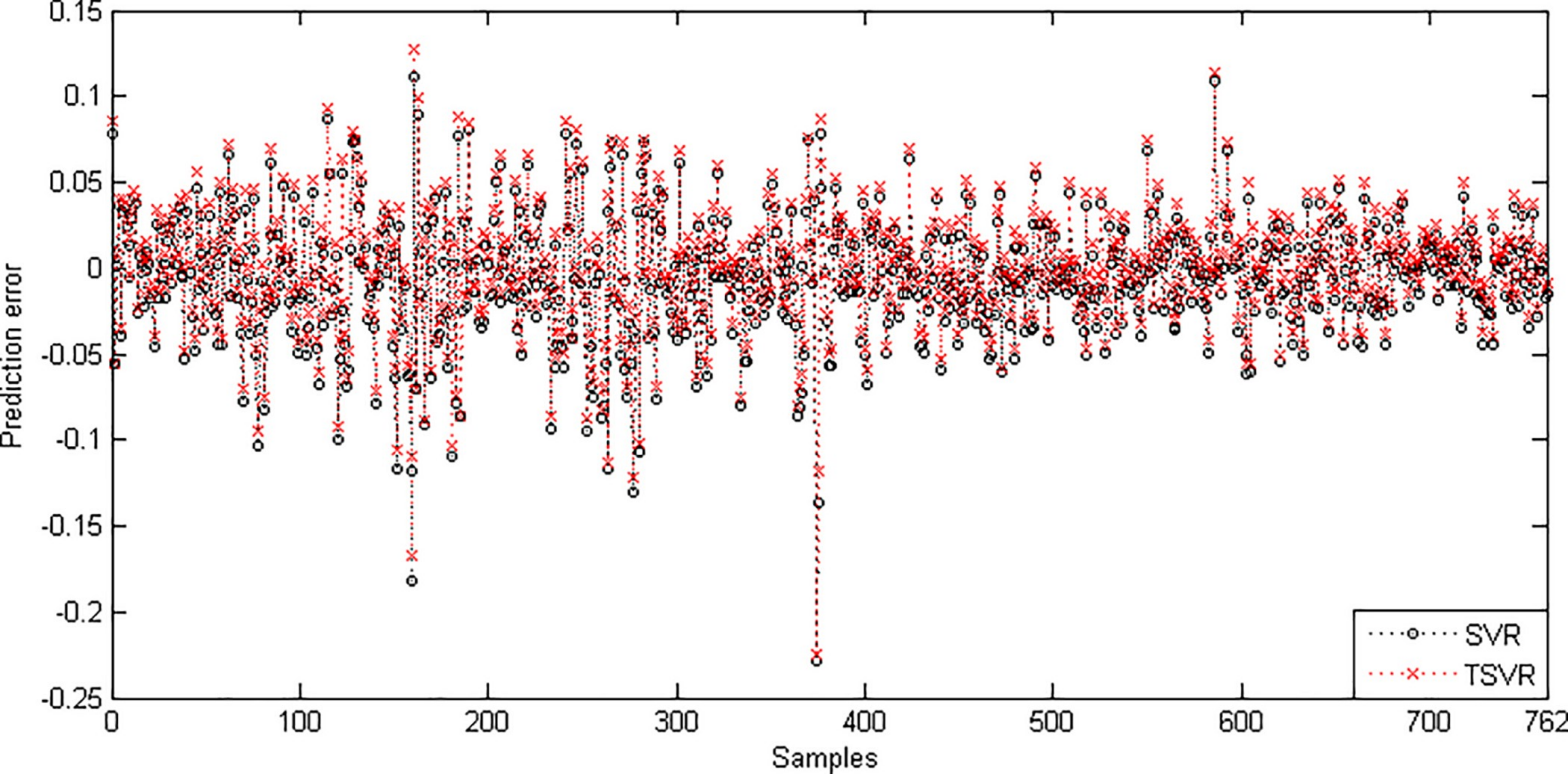
Prediction error plots using a linear kernel on the BFX dataset.

**Fig 8:**
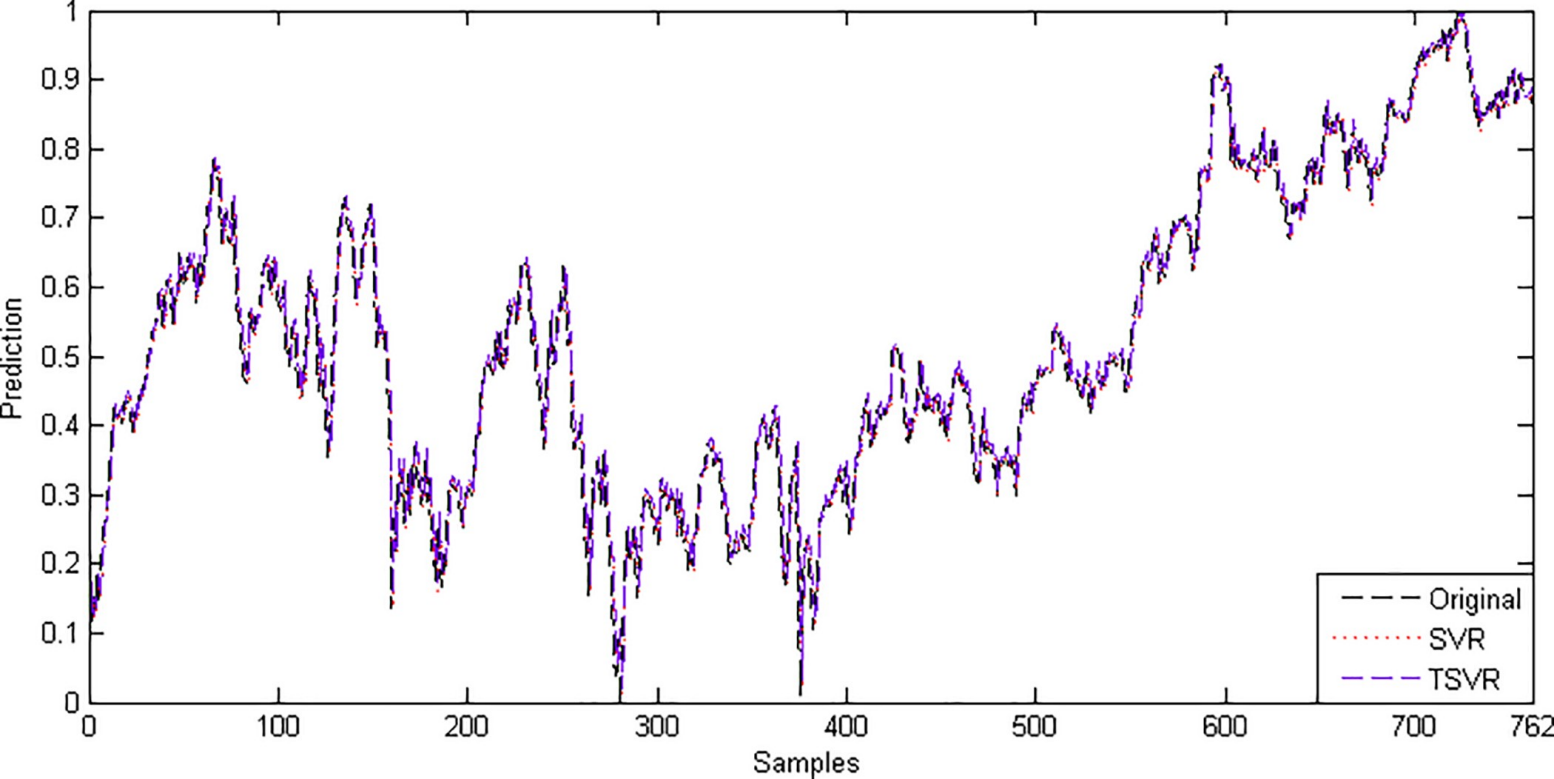
Predicted and actual values using a linear kernel on the BFX dataset.

For the linear kernel, Table 6 shows the average RMSE for the optimal parameter values with the standard deviation and the training time in seconds. We can conclude that TSVR gives better results in 13 cases out of 20 datasets in terms of average RMSE of test accuracy. Additionally, the training time of TSVR is lower than that of SVR. The Friedman statistical nonparametric post hoc test is performed on the average rank of 20 financial datasets from Table 7. The Friedman statistic [40] can be computed under the null hypothesis for the linear case:

$$\chi_{F}^{2}=\frac{12\times 20}{2\times (2+1)}\left[(1.65^{2}+1.35^{2})-\frac{2\times {\left(2+1\right)}^{2}}{4}\right]≅1.80$$

$$F_{F}=\frac{(20-1)\times 1.8}{20\times (2-1)-1.8}≅1.8791$$

where *F*_*F*_ is distributed according to the *F*-distribution with (1,19), which has the critical value 4.3807 for the level of significance *α* = 0.05. Here, *F*_*F*_ is less than the critical value, so there is no significant difference between these two algorithms for the linear case. Fig 7 shows the absolute prediction error plot of SVR and TSVR for the linear kernel on the BFX dataset. Fig 8 also shows the actual and predicted values of SVR and TSVR for the linear kernel on the market stock index BFX dataset. One can easily conclude that TSVR is in close agreement with the target values compared to SVR.

**Table 6:** Performance comparison of TSVR with SVR on stock market index datasets using a linear kernel. RMSE is used for comparison. Time is used for the training in seconds.

Dataset	SVR (C) Time	TSVR (C1 = C2) Time
**AEX** **(763X6)**	0.02683+0.01051 (10^-1) 11.8233	0.02678+0.01061 (10^5) 1.47306
**ATX** **(737X6)**	0.01886+0.00414 (10^-2) 10.3641	0.01885+0.0043 (10^1) 1.1216
**BFX** **(762X6)**	0.03424+0.01144 (10^-1) 11.3085	0.03545+0.01039 (10^3) 1.16305
**BSESN** **(733X6)**	0.02062+0.00448 (10^-1) 10.2492	0.02071+0.00445 (10^1) 1.22084
**BVSP** **(738X6)**	0.01993+0.00365 (10^-2) 10.4724	0.01997+0.00379 (10^2) 0.97825
**DJI** **(751X6)**	0.01413+0.00492 (10^-1) 10.8441	0.01419+0.0048 (10^2) 1.39238
**FCHI** **(763X6)**	0.03166+0.01213 (10^-2) 11.1665	0.03159+0.01216 (10^2) 0.93741
**GDAXI** **(755X6)**	0.02591+0.00872 (10^-2) 10.8492	0.02586+0.00873 (10^1) 1.12026
**GSPTSE** **(748X6)**	0.02208+0.00768 (10^-1) 10.6209	0.02214+0.00779 (10^2) 1.28185
**HSI** **(733X6)**	0.02125+0.00607 (10^-2) 10.2733	0.0212+0.00608 (10^1) 1.26684
**IBEX** **(763X6)**	0.02829+0.00918 (10^-2) 11.1037	0.02828+0.0091 (10^1) 1.44011
**IXIC** **(751X6)**	0.0165+0.00475 (10^-1) 10.8561	0.01645+0.00473 (10^2) 1.10158
**JKSE** **(724X6)**	0.01871+0.0053 (10^-1) 10.4427	0.18938+0.36737 (10^5) 1.19995
**KS11** **(728X6)**	0.02053+0.00366 (10^-2) 10.1628	0.02052+0.00367 (10^2) 0.90443
**MXX** **(750X6)**	0.03059+0.00594 (10^-1) 10.6947	0.03052+0.006 (10^1) 1.47527
**N225** **(732X6)**	0.02757+0.01059 (10^-1) 10.2778	0.02753+0.01071 (10^1) 1.14582
**NSEI** **(731X6)**	0.01992+0.00419 (10^-1) 10.1286	0.01994+0.00419 (10^1) 1.18078
**SSMI** **(750X6)**	0.0402+0.0164 (10^-1) 10.8077	0.04008+0.01626 (10^1) 1.32179
**STOXX50E** **(745X6)**	0.032+0.01324 (10^-2) 10.5735	0.03193+0.01327 (10^1) 1.1432
**TWII** **(724X6)**	0.02051+0.00474 (10^-1) 10.0368	0.02049+0.00477 (10^2) 1.23588

**Table 7:** Average ranks of TSVR with SVR on stock market index datasets using a linear and Gaussian kernel.

Dataset	Linear		Non-Linear	
	SVR	TSVR	SVR	TSVR
**AEX**	2	1	2	1
**ATX**	2	1	2	1
**BFX**	1	2	2	1
**BSESN**	1	2	2	1
**BVSP**	1	2	2	1
**DJI**	1	2	2	1
**FCHI**	2	1	2	1
**GDAXI**	2	1	2	1
**GSPTSE**	1	2	2	1
**HIS**	2	1	2	1
**IBEX**	2	1	2	1
**IXIC**	2	1	2	1
**JKSE**	1	2	2	1
**KS11**	2	1	2	1
**MXX**	2	1	2	1
**N225**	2	1	2	1
**NSEI**	1	2	2	1
**SSMI**	2	1	1	2
**STOXX50E**	2	1	2	1
**TWII**	2	1	2	1
**Average rank**	1.65	1.35	1.95	1.05

#### Nonlinear case

For the non-linear kernel, Table 8 shows the average RMSE for the optimal parameter value with the standard deviation and the training time in seconds. We can conclude that TSVR gives better results in 19 out of 20 datasets in terms of average RMSE of test accuracy. The training time of TSVR is less than that of SVR due to solving a pair of smaller-sized QPPs instead of a large QPP, as in the case of SVR. This shows the superiority of TSVR with respect to SVR.

**Table 8:** Performance comparison of TSVR with SVR on stock market index datasets using a Gaussian kernel. RMSE is used for comparison. Time is used for the training in seconds.

**AEX** **(763X6)**	0.02765+0.01023 (10^0,2^-2) 12.781	0.02698+0.0106 (10^2,2^-5) 1.73396
**ATX** **(737X6)**	0.01949+0.00416 (10^-1,2^-2) 11.907	0.01892+0.00422 (10^2,2^-5) 1.41487
**BFX** **(762X6)**	0.03466+0.01048 (10^-2,2^-1) 12.787	0.03395+0.0117 (10^2,2^-5) 1.40597
**BSESN** **(733X6)**	0.02264+0.00551 (10^0,2^-2) 11.8247	0.02073+0.00453 (10^3,2^-4) 1.56612
**BVSP** **(738X6)**	0.0222+0.00447 (10^-1,2^-5) 11.9909	0.02005+0.00391 (10^2,2^-5) 1.43526
**DJI** **(751X6)**	0.01721+0.00561 (10^0,2^-5) 12.3971	0.0155+0.00503 (10^2,2^-5) 1.74354
**FCHI** **(763X6)**	0.03171+0.01203 (10^-1,2^-2) 12.6618	0.03156+0.01218 (10^2,2^-5) 1.53077
**GDAXI** **(755X6)**	0.02662+0.00822 (10^-1,2^-2) 12.4439	0.02601+0.00867 (10^2,2^-5) 1.49756
**GSPTSE** **(748X6)**	0.02627+0.0173 (10^-1,2^-2) 12.2009	0.02301+0.00875 (10^3,2^-5) 1.44924
**HSI** **(733X6)**	0.02189+0.00633 (10^-1,2^-2) 11.7218	0.02156+0.00623 (10^2,2^-5) 1.38225
**IBEX** **(763X6)**	0.0285+0.00925 (10^-1,2^-4) 12.6977	0.02842+0.00915 (10^2,2^-5) 1.47464
**IXIC** **(751X6)**	0.01906+0.00513 (10^0,2^-5) 12.3108	0.01681+0.0047 (10^2,2^-5) 1.69016
**JKSE** **(724X6)**	0.01922+0.00522 (10^0,2^-2) 11.3828	0.01893+0.00533 (10^2,2^-2) 1.57983
**KS11** **(728X6)**	0.02197+0.00448 (10^-1,2^-4) 11.6135	0.02073+0.00373 (10^2,2^-5) 1.34512
**MXX** **(750X6)**	0.03145+0.00605 (10^0,2^-5) 12.4093	0.03082+0.0058 (10^2,2^-3) 1.73352
**N225** **(732X6)**	0.02952+0.01077 (10^-1,2^-5) 12.0234	0.02839+0.01029 (10^2,2^-5) 1.3794
**NSEI** **(731X6)**	0.02206+0.00591 (10^-2,2^-1) 11.9166	0.02013+0.00444 (10^3,2^-5) 1.29858
**SSMI** **(750X6)**	0.04002+0.01628 (10^-1,2^-4) 12.3919	0.04007+0.0161 (10^2,2^-5) 1.42911
**STOXX50E** **(745X6)**	0.03218+0.01336 (10^0,2^-4) 12.4306	0.03204+0.01328 (10^2,2^-5) 1.66912
**TWII** **(724X6)**	0.02084+0.0046 (10^-1,2^-1) 11.495	0.02057+0.00472 (10^2,2^-5) 1.34332

In the nonlinear case for different stock market index datasets, the Friedman statistic can be computed under the null hypothesis from Table 7 as:

$$\chi_{F}^{2}=\frac{12\times 20}{2\times (2+1)}\left[(1.95^{2}+1.05^{2})-\frac{2\times {\left(2+1\right)}^{2}}{4}\right]≅16.2$$

$$F_{F}=\frac{(20-1)\times 16.2}{20\times (2-1)-16.2}≅81$$

where *F*_*F*_ is the distribution according to the *F*-distribution with (1,1×19) = (1,19) as the degree of freedom. Here, 4.3807 is the critical value of *F*(1,19) for the level of significance at *α* = 0.05. Since the value of *F*_*F*_ = 81>4.3807 is rejected, we reject the null hypothesis. Similar to the previous case, we perform pairwise comparisons using the Nemenyi post hoc test for all reported methods and verify the significant critical difference between their average ranks. The difference between their ranks should be at least $1.645\sqrt{\frac{2\times (2+1)}{6\times 20}}\approx 0.3678$ at *p* = 0.10.

**Fig 9:**
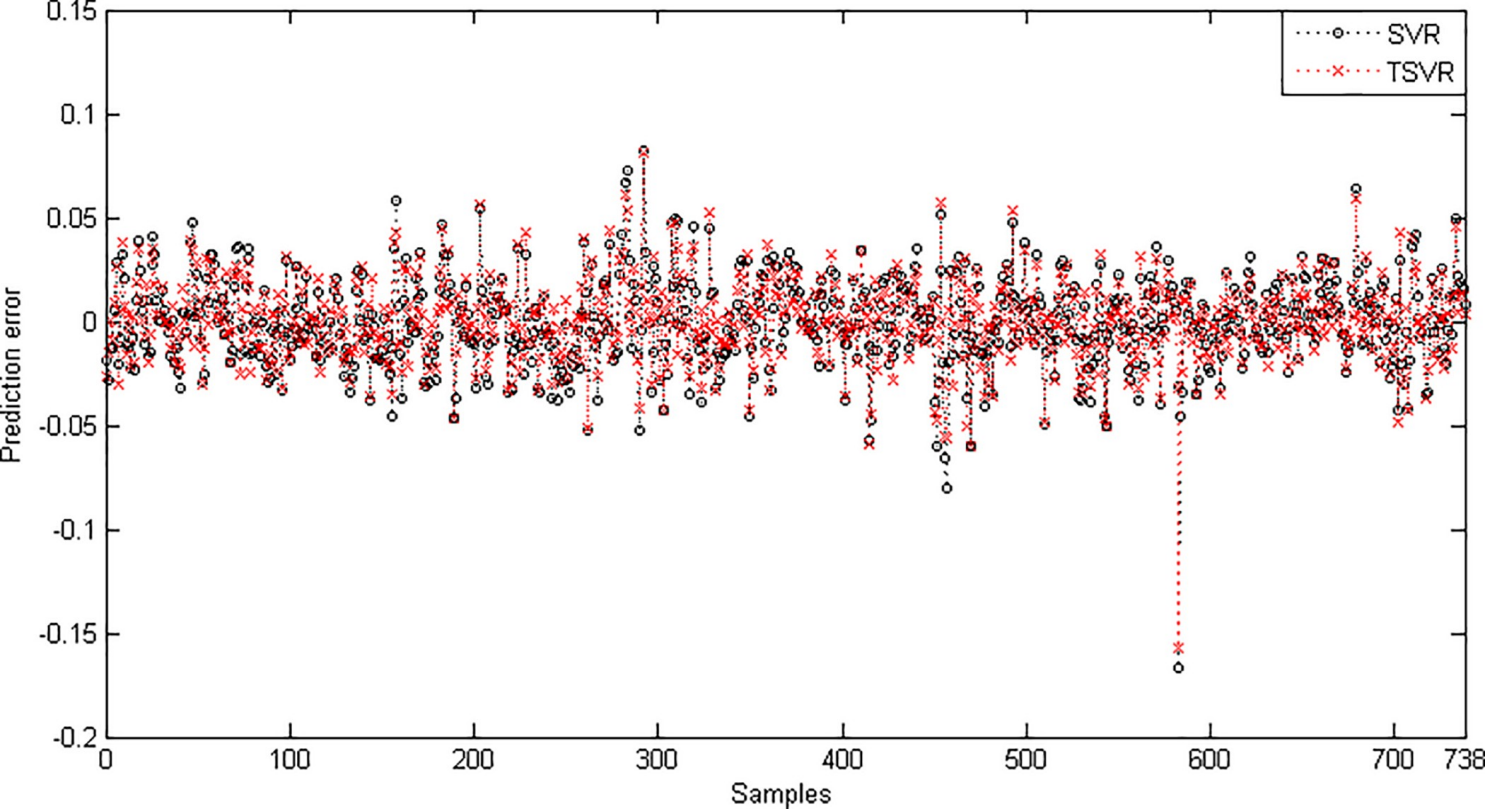
Prediction error plots using a Gaussian kernel on the BVSP dataset.

**Fig 10:**
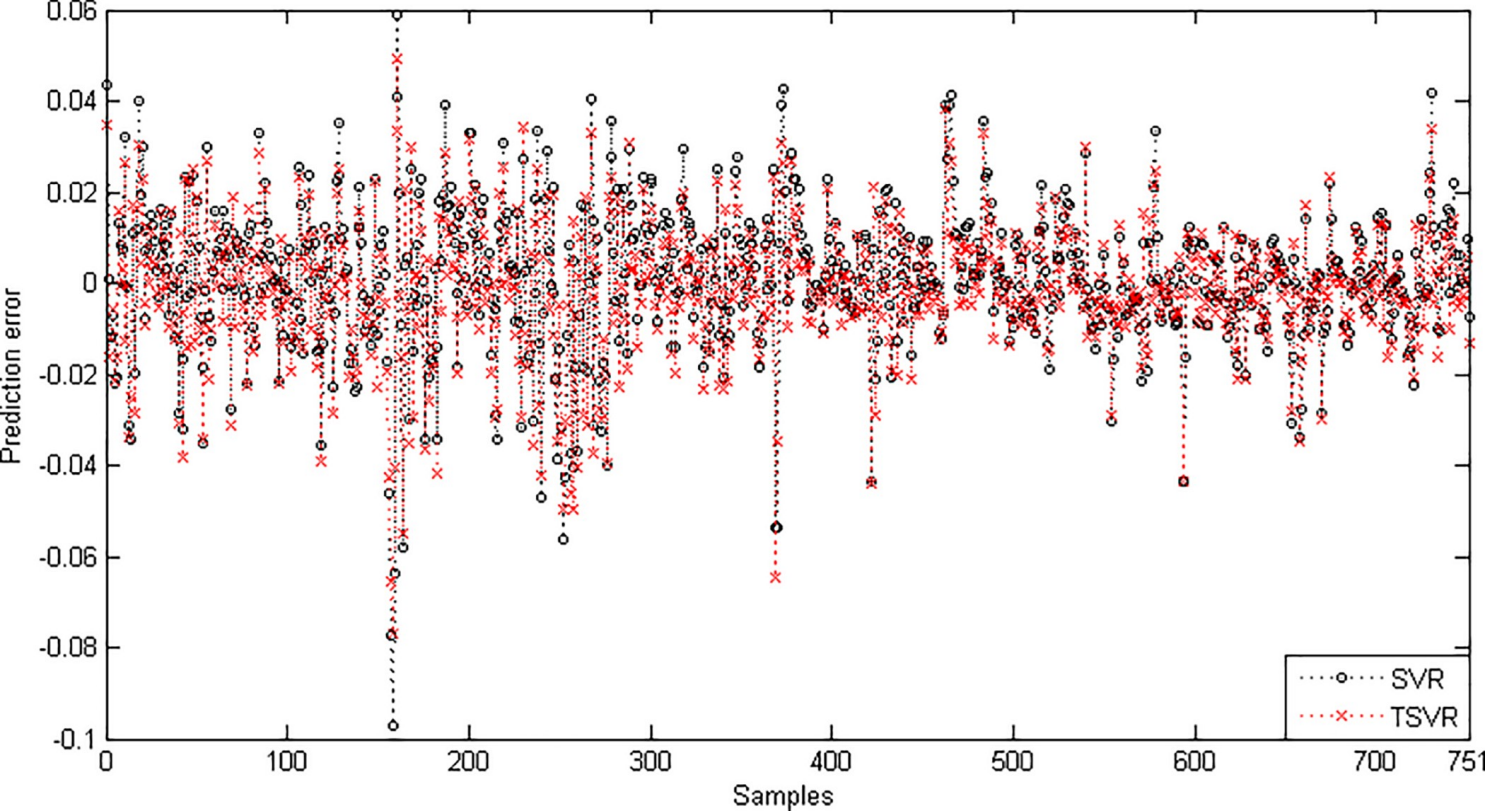
Prediction error plots using a Gaussian kernel on the DJI dataset.

**Fig 11:**
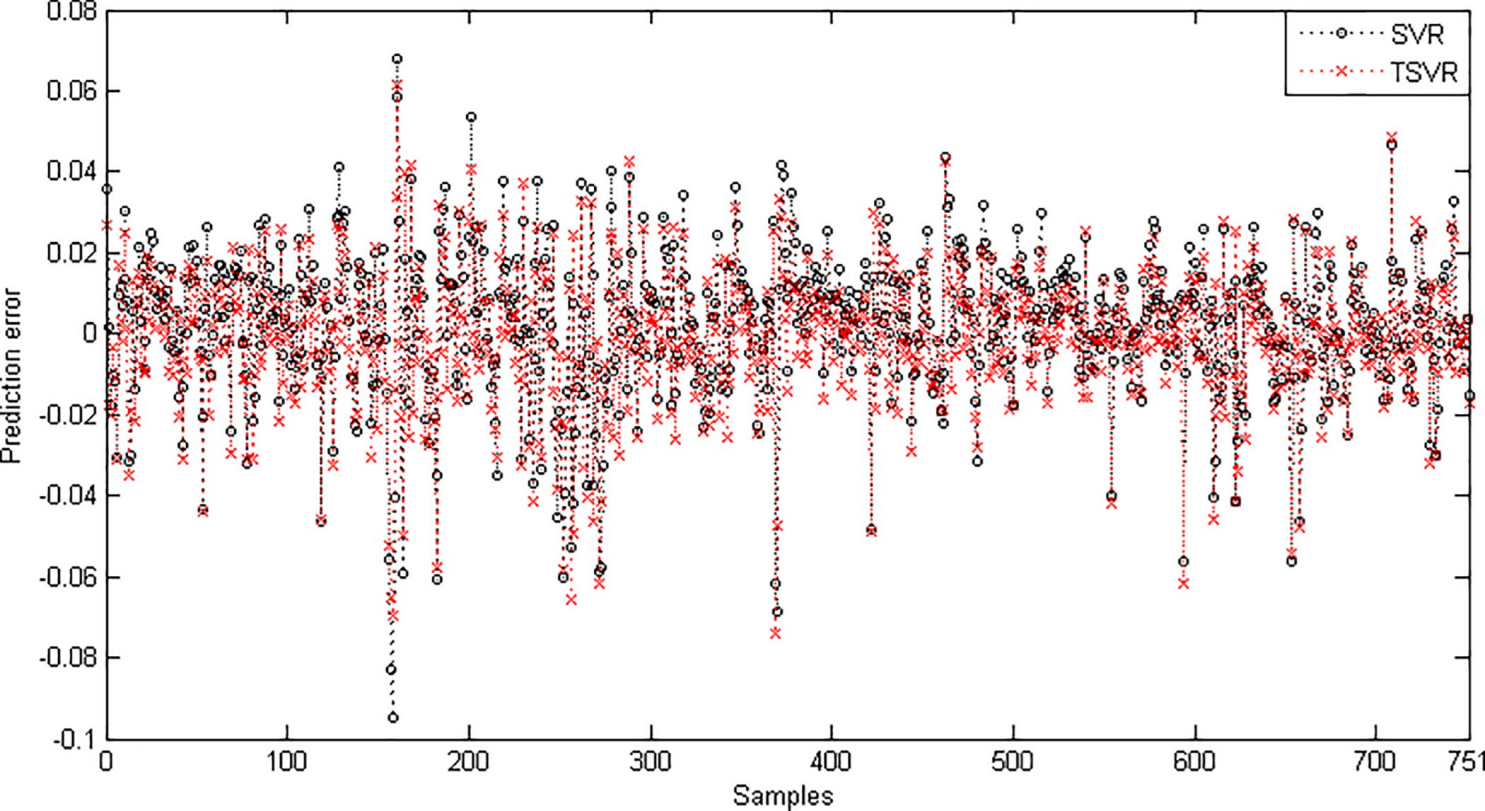
Prediction error plots using a Gaussian kernel on the IXIC dataset.

**Fig 12:**
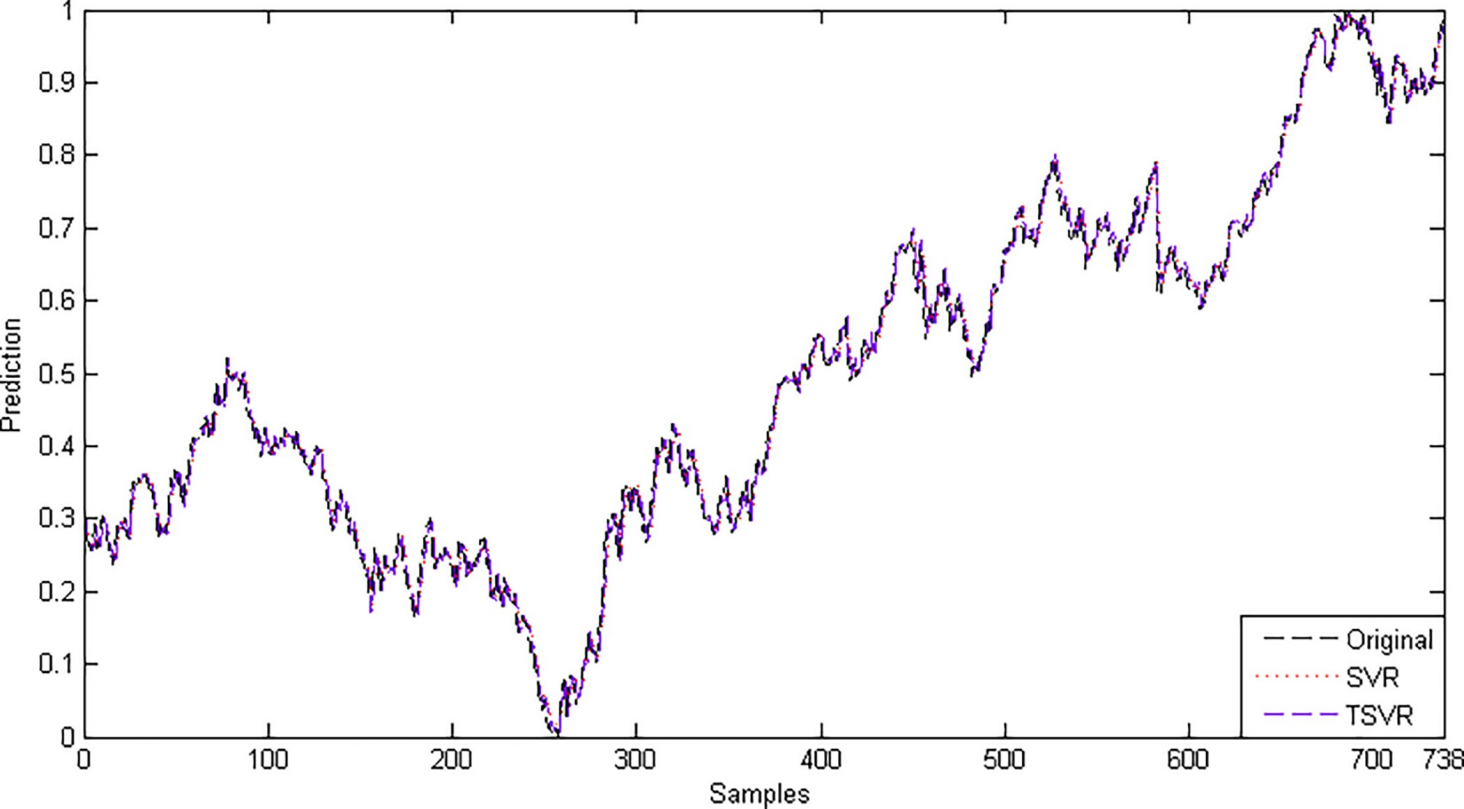
Predicted and actual values using a Gaussian kernel on the BVSP dataset.

**Fig 13:**
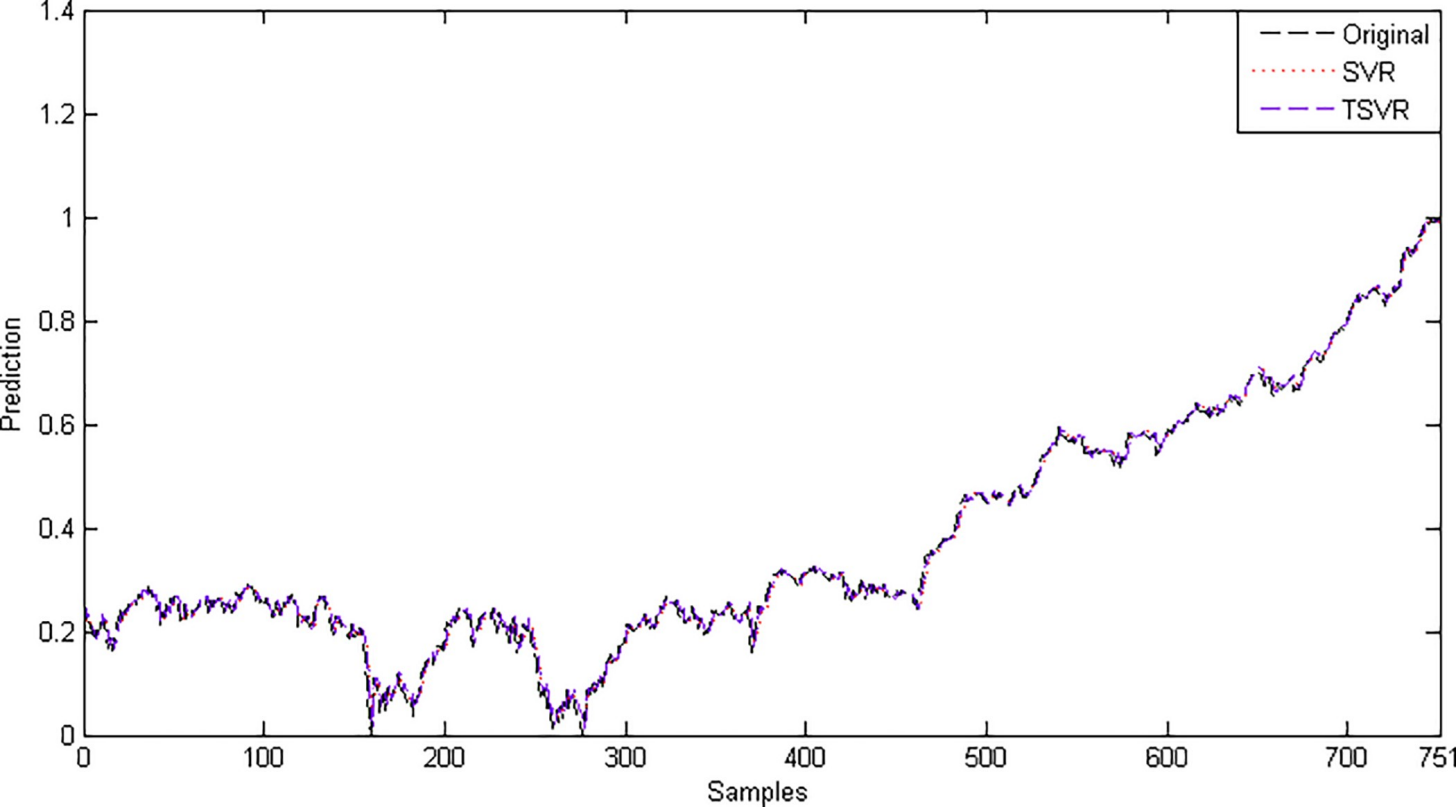
Predicted and actual values using a Gaussian kernel on the DJI dataset.

**Fig 14:**
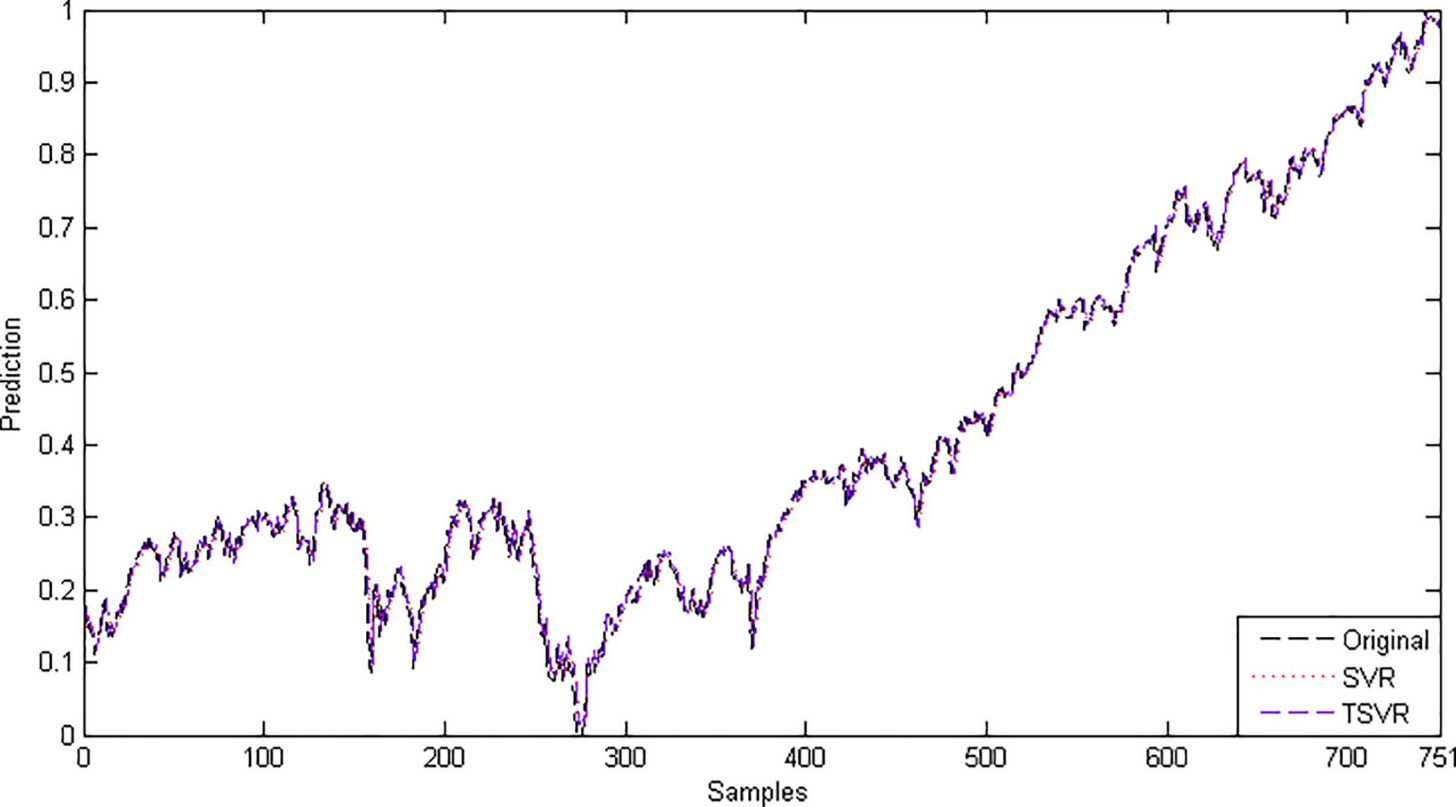
Predicted and actual values using a Gaussian kernel on the IXIC dataset.

Since the difference between the average ranks of TSVR with SVR (1.95−1.05 = 0.90) is greater than 0.3678, we conclude that TSVR is significantly better than SVR for stock market index datasets. For the non-linear case, the absolute prediction error of SVR and TSVR is shown in Figs 9, 10 and 11 for the BVSP, DJI and IXIC datasets, respectively. The actual and predicted values of SVR and TSVR are plotted in Figs 12, 13 and 14 for the BVSP, DJI and IXIC datasets, respectively. It can easily be observed from these figures that TSVR is in close agreement with the desired output in comparison to SVR, which clearly demonstrates the applicability and usefulness of TSVR.

## Conclusion

In this paper, support vector regression and twin support vector regression formulations are discussed in detail and applied to an individual companies’ stock indices in the area of information technology industries, banking, oil, and petroleum industry and stock market index datasets of different countries to predict stock prices. Here, a pair of smaller sized QPPs is solved instead of a single large sized QPP, as in the case of SVR, thus yielding a reduction in the cost of the system. To verify the effectiveness of TSVR, we performed numerical experiments for both linear and Gaussian kernels on financial time series datasets. In experimental results, TSVR shows better learning speed for both linear and Gaussian kernels with the ability to predict having a better generalization ability than SVR. In fact, the computation time of the TSVR is approximately four times lower than the standard SVR in terms of learning speed, which clearly indicates its existence and usability. In future work, a new model that is able to handle noise and outliers for predicting the prices of stock indices can be explored.
